# An AIC-type information criterion evaluating theory-based hypotheses for contingency tables

**DOI:** 10.3758/s13428-024-02570-6

**Published:** 2025-01-22

**Authors:** Yasin Altinisik, Roy S. Hessels, Caspar J. Van Lissa, Rebecca M. Kuiper

**Affiliations:** 1 Department of Statistics, Sinop University, Osmaniye Mahallesi, Selanik Caddesi (Kuzey Kampüs), No:52G, 57000 Sinop, Türkiye; 2 Experimental Psychology, Helmholtz Institute, Utrecht University, Utrecht, The Netherlands; 3 Department of Methodology and Statistics, Tilburg University, Tilburg, The Netherlands; 4 Department of Methodology and Statistics, Utrecht University, Utrecht, The Netherlands

**Keywords:** AIC, Contingency tables, GORICA, (In)equality constraints, Theory-based hypotheses

## Abstract

Researchers face inevitable difficulties when evaluating theory-based hypotheses in the context of contingency tables. Log-linear models are often insufficient to evaluate such hypotheses, as they do not provide enough information on complex relationships between cell probabilities in many real-life applications. These models are usually used to evaluate the relationships between variables using only equality restrictions between model parameters, while specifying theory-based hypotheses often also requires inequality restrictions. Moreover, high-dimensional contingency tables generally contain low cell counts and/or empty cells, complicating parameter estimation in log-linear models. The presence of many parameters in these models also causes difficulties in interpretation when evaluating the hypotheses of interest. This study proposes a method that simplifies evaluating theory-based hypotheses for high-dimensional contingency tables by simultaneously addressing each of the above problems. With this method, theory-based hypotheses, which are specified using equality and/or inequality constraints with respect to (functions of) cell probabilities, are evaluated using an AIC-type information criterion, GORICA. We conduct a simulation study to evaluate the performance of GORICA in the context of contingency tables. Two empirical examples illustrate the use of the method.

**Supplementary Information:**

The online version contains supplementary material available at 10.3758/s13428-024-02570-6.

## Introduction

Contingency tables can be utilized to investigate the relationships between two or more nominal or ordinal variables. An important model that can be used to analyze contingency tables is the log-linear model (Agresti, 2007; Azen & Walker, 2010). In this model, a linear combination of predictors is related to the cell probabilities using a log-link function. However, when the contingency table is high-dimensional, it might be virtually impossible to interpret the log-linear parameters representing the associations between the predictors and cell probabilities in the model (Hendrickx, 2004, p.603). For example, consider a $5 \times 5 \times 5$ contingency table modeled using a full log-linear model. This model contains 124 parameters in total, that is, 12 main effects, 48 two-way interaction effects, and 64 three-way interaction effects. There are many parameters in this model, and it is obvious that the three-way interaction effects are hard to interpret. In addition, high-dimensional contingency tables often have empty cells, causing unreliable estimates or inestimable parameters in log-linear models. To perform inference on contingency tables by interpreting the estimates in log-linear models, often the hypothesis of no association is tested, while researchers generally expect some association between the variables.

An alternative to null-hypothesis testing is to evaluate a theory-based hypothesis that represents researchers’ expectations about the association between nominal and ordinal variables (using model selection). Theory-based hypotheses can be expressed using equality and/or inequality restrictions. To illustrate theory-based hypotheses in the context of contingency tables, consider the study of Moore et al. (2009, p. 305). They investigated the degrees earned in higher education in terms of gender in the USA during the academic year 2010–2011. The nominal variable gender is defined as G $\in$ {1 = Female, 2 = Male}. The ordinal variable academic degree is defined as AD $\in$ {1 = Bachelor’s, 2 = Master’s, 3 = Professional, 4 = Doctorate}. The population probability of a person having the *i*th level of gender and the *j*th level of academic degree is represented by $\pi _{ij}$ with $i = 1, 2$ and $j = 1, 2, 3, 4$. The population probabilities and the observed cell frequencies are displayed in Table 1. In this study, a hypothesis of interest could be: $H_{1}:$ The proportion of males in bachelor’s degree holders is larger than that in any other degree holders. This hypothesis can be represented by the theory-based hypothesis:

1 $$\begin{aligned} H_{1}: \frac{\pi _{21}}{\pi _{+1}} > \{\frac{\pi _{22}}{\pi _{+2}},\frac{\pi _{23}}{\pi _{+3}},\frac{\pi _{24}}{\pi _{+4}}\}, \end{aligned}$$

where the operator “+” denotes a sum of cell probabilities over the levels of the corresponding variable for one level of the other variable(s). For example, $\pi _{+1} = \pi _{11} + \pi _{21}$ denotes the sum of probabilities over the levels of gender (i.e., men and women) for the first level of academic degree (i.e., bachelor’s degree). Stated otherwise, $\pi _{+1}$ is the sum of proportions of the bachelor’s degrees for females and males and denotes the prevalence of a bachelor’s degree.

**Table 1:** Population probabilities $\pi _{ij}$ and observed cell frequencies between brackets for the combinations of gender and academic degree

$\pi _{ij}$	1 = Bachelor’s	2 = Master’s	3 = Professional	4 = Doctorate
1 = Female	$\pi _{11}$ (933)	$\pi _{12}$ (402)	$\pi _{13}$ (51)	$\pi _{14}$ (26)
2 = Male	$\pi _{21}$ (661)	$\pi _{22}$ (260)	$\pi _{23}$ (44)	$\pi _{24}$ (26)

This paper proposes a method to evaluate the hypotheses of interest using the contingency table instead of the log-linear model. First, a set of theory-based hypotheses should be formulated. Second, an AIC-type information criterion (Akaike, 1973, 1974) is used to quantify the support in the data for these hypotheses, which is called the generalized order-restricted information criterion approximation (GORICA; Altinisik et al., 2021). GORICA has a simple and straightforward interpretation and can be used for analyzing high-dimensional sparse contingency tables, that is, high-dimensional contingency tables with low cell counts and/or empty cells.

GORICA (Altinisik et al., 2021) is an AIC-type information criterion that can be used to evaluate theory-based hypotheses for generalized linear models (GLMs; McCullagh and Nelder, 1989), generalized linear mixed models (GLMMs; McCullogh and Searle, 2001), and structural equation models (SEMs; Bollen, 1989).^1^. GORICA can be used to evaluate linear restrictions on model parameters in these classes of models. However, as is exemplified by hypothesis $H_{1}$ in (1), hypotheses in the context of contingency tables are often expressed through non-linear restrictions on cell probabilities. In this paper, we elaborate on a) how GORICA can evaluate hypotheses containing linear or non-linear restrictions on cell probabilities and b) how GORICA can easily be applied to high-dimensional contingency tables with or without empty cells. The algorithms described in this paper have been implemented in the existing gorica package (Van Lissa et al., 2020), in user-friendly functions that accept an R contingency table, generated by table() as input.

In this paper, we first introduce GORICA for theory-based hypotheses that contain only linear restrictions. Second, we explain the general idea of computing the penalty part of GORICA when hypotheses contain linear restrictions on cell probabilities. Third, we discuss five classes of restrictions that are relevant for hypotheses regarding contingency tables, where four of them contain non-linear restrictions. Fourth, we elaborate on two possible problems associated with evaluating theory-based hypotheses using GORICA, which are non-linear restrictions on cell probabilities and empty cells in contingency tables. We offer solutions to both problems and have incorporated them into our accompanying R package. Fifth, we investigate the performance of GORICA in the context of contingency tables when hypotheses contain non-linear restrictions on cell probabilities in the presence of empty cell(s). Sixth, GORICA is applied to two examples. The first example, based on the gender by earned degrees example above, involves evaluating hypotheses containing non-linear restrictions on cell probabilities. The second example concerns the evaluation of hypotheses containing linear restrictions on cell probabilities for two high-dimensional contingency tables with empty cells. The paper closes with a discussion.

## GORICA

The generalized order-restricted information criterion appro-ximation (GORICA; Altinisik et al., 2021) is an AIC-type information criterion that can be used to evaluate theory-based hypotheses. GORICA is an extension of the generalized order-restricted information criterion (GORIC; Kuiper et al., 2011, 2012). GORIC can be used in the context of normal linear models, whereas GORICA can evaluate hypotheses for more families of statistical models, namely, for generalized linear models (GLMs; McCullagh and Nelder, 1989), generalized linear mixed models (GLMMs; McCullogh and Searle, 2001), and structural equation models (SEMs; Bollen, 1989). Similar to GORIC, GORICA can be used to evaluate multiple hypotheses simultaneously. The last hypothesis in the set of hypotheses under evaluation is often the unconstrained hypothesis $H_{u}$, sometimes called the traditional alternative hypothesis. This hypothesis can be included in the set of theory-based hypotheses to avoid that the best hypothesis is nevertheless a weak hypothesis, thus serving as a failsafe.^2^

Like other information criteria, GORICA evaluates hypo-theses based on their fit and complexity. The fit is quantified by the maximum of the log-likelihood under the restriction(s) of hypothesis $H_{m}$. The complexity penalizes hypothesis $H_{m}$ based on its restrictions on model parameters and can be seen as the expected number of free parameters in the hypothesis. GORICA selects the best hypothesis from a set of competing hypotheses, that is, the hypothesis that has the smallest distance in terms of the Kullback–Leibler divergence (Kullback & Leibler, 1951) to the true hypothesis relative to the other hypotheses in the set. GORICA value of hypothesis $H_{m}$ is:

2 $$\begin{aligned} \textrm{GORICA}_{m} = -2\text L(\varvec{\tilde{\theta }}_{m}|\varvec{\hat{\theta }}, \varvec{\hat{\Sigma }}_{\varvec{\hat{\theta }}})+ 2PT_{m}(\varvec{\theta }), \end{aligned}$$

where $L(\varvec{\tilde{\theta }}_{m}|\varvec{\hat{\theta }}, \varvec{\hat{\Sigma }}_{\varvec{\hat{\theta }}})$ denotes the order-restricted maximum log-likelihood and $PT_{m}(\varvec{\theta })$ denotes the penalty. Here, the $\varvec{\hat{\theta }}\in \mathrm I\!R^{K \times 1}$ are the maximum likelihood estimates (MLEs) of $\varvec{\theta }$ (with *K* representing the number of parameters used in the hypotheses). The $\varvec{\hat{\Sigma }}_{\varvec{\hat{\theta }}} \in \mathrm I\!R^{K \times K}$ is the covariance matrix of the MLEs. When hypotheses contain (the functions of) cell probabilities, the covariance matrix of the MLEs, $\varvec{\hat{\Sigma }}_{\varvec{\hat{\theta }}} \in \mathrm I\!R^{K \times K}$, are obtained using nonparametric bootstrapping (see Appendix A). The order-restricted MLEs $\varvec{\tilde{\theta }}_{m} \in \mathrm I\!R^{K \times 1}$ represent the MLEs after the (in)equality constraints in $H_{m}$ are imposed on the $\theta$ parameters, that is, the MLEs that are in agreement with the constraints in $H_{m}$. The order-restricted MLEs, $\varvec{\tilde{\theta }}_{m} \in \mathrm I\!R^{K \times 1}$, are obtained using a quadratic non-linear programming algorithm (see the end of Appendix A).

The parameter vector $\varvec{\theta }$ is used for the general formulation of GORICA, and it can take different forms. For example, the $\theta$s are defined in the real plane (i.e., $\varvec{\theta }\in \mathrm I\!R^{K \times 1}$) when the hypotheses of interest contain restrictions on the parameters of a log-linear model. As will be shown later, when hypotheses involve linear restrictions on cell probabilities, the $\theta$s range between 0 and 1 and they sum to 1 (i.e., $\varvec{\theta }= \varvec{\pi }$ with $0< \varvec{\pi }< 1$ and $\sum _{k = 1}^{K} \pi _{k} = 1$). When hypotheses contain conditional cell probabilities, the $\theta$s are between 0 and 1 and they may or may not sum to 1 (i.e., $\varvec{\theta }= \varvec{\eta }$ with $0< \varvec{\eta }< 1$). If the hypotheses contain odds ratios, the $\theta$s are positive for the complete data (i.e., $\varvec{\theta }= \varvec{\eta }$ with $\varvec{\eta }> 0$).

**Fig. 1:**
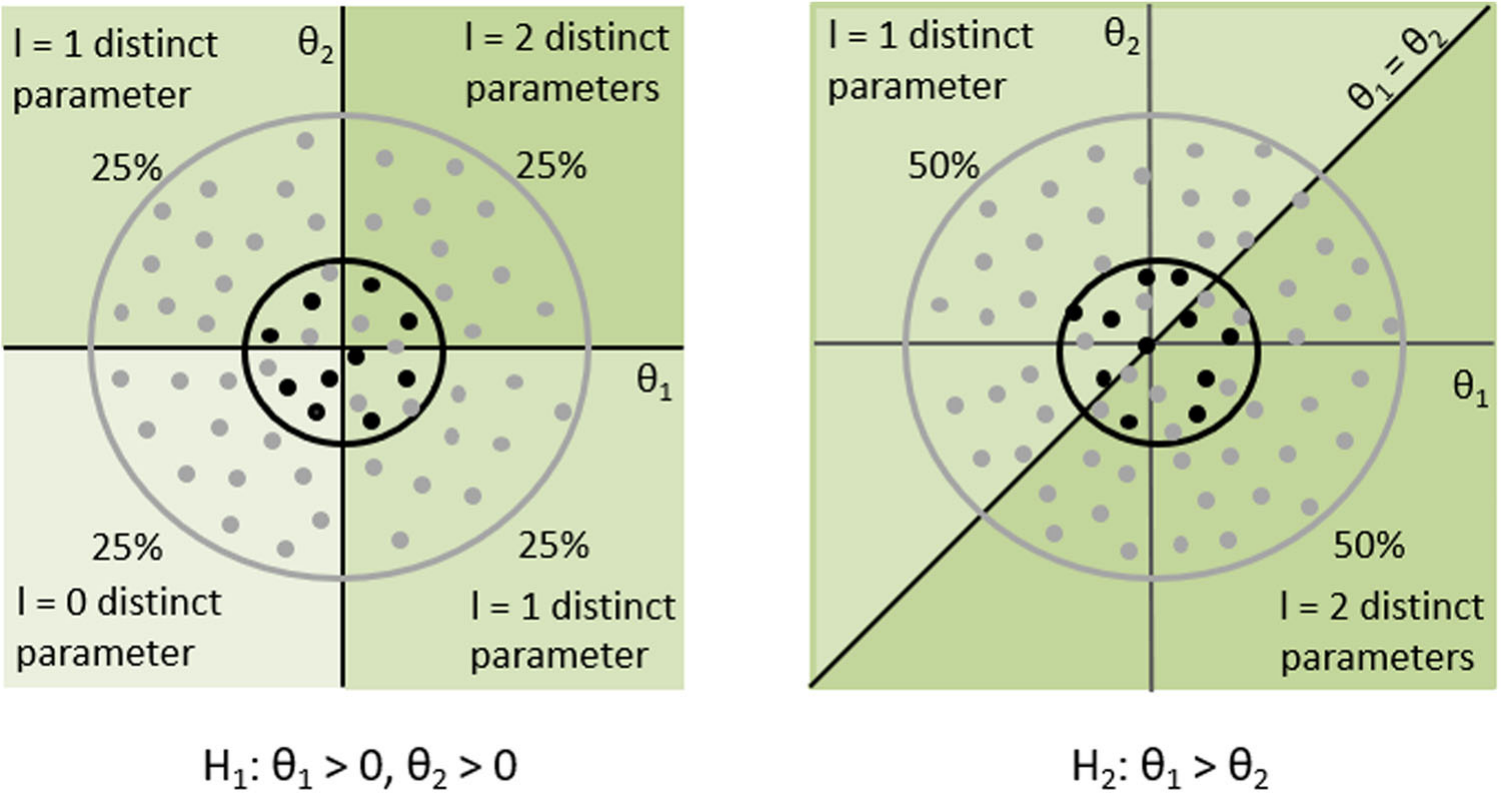
Penalty calculation for closed convex cones with different sizes of inspected spaces (denoted by *circles*)

Regardless of the form of parameter vector $\varvec{\theta }$ above, the penalty part of GORICA for hypothesis $H_{m}$, $PT_{m}(\varvec{\theta })$ in (2), can be calculated based on a null distribution for $\varvec{\theta }$ (Silvapulle & Sen, 2005). The null distribution is a normal distribution with a mean vector of zeros, $\varvec{\mu }$, and the covariance matrix of MLEs, $\varvec{\hat{\Sigma }}_{\varvec{\hat{\theta }}}$ (Altinisik et al., 2021, p. 621). Once the GORICA values are calculated, they can be transformed into the GORICA weights (Altinisik et al., 2021, p. 603), which quantify the support in the data for each hypothesis under evaluation compared to the others in the set (see Appendix B).

## Computing the penalty

The penalty value of hypothesis $H_{m}$ represents the complexity of this hypothesis relative to the other hypotheses in the set. It can be seen as the expected number of free parameters^3^ after taking into account the restrictions in hypothesis $H_{m}$. The penalty is defined as:

3 $$\begin{aligned} PT_{m}(\varvec{\theta }) = \sum _{\textit{l }= 1}^{K}\textrm{LP}_{\textit{l}} \times \textit{l}, \end{aligned}$$

where $\textrm{LP}_{l}(.)$ represents the *l*th level probability (LP) for $l = 1, 2, ..., K$. The LPs are determined using a null distribution for $\varvec{\theta }$, which is a normal distribution with a mean vector of zeros, $\varvec{\mu }$, and the covariance matrix of MLEs, $\varvec{\hat{\Sigma }}_{\varvec{\hat{\theta }}}$. The details of computing the penalty using LPs are explained in Altinisik et al. (2021, p. 621). Next, we elaborate on the general idea of computing the penalty. Note that we use the general $\theta$ parameters, that is, the $\theta$s that can take on any real value, to show how the penalty is calculated in different cases. We also utilize figures to depict the penalty calculation in these instances.

To determine the LPs, we sample from the null distribution shown as gray and black dots in Fig. 1. The dark green area indicates the feasible region of $\varvec{\theta }$, that is, the parameter space that is in agreement with the restrictions of the hypothesis of interest. When we inspect $H_{1}: \theta _{1}> 0, \theta _{2} > 0$, half of the samples are in accordance with either $\theta _{1} > 0$ or $\theta _{2} > 0$ corresponding to one free parameter (i.e., $\textrm{LP}_{1} = 0.25 + 0.25 = 0.50$). A quarter of the samples are in agreement with both restrictions corresponding to two free parameters (i.e., $\textrm{LP}_{2} = 0.25$). Note that a quarter of the samples are inconsistent with either restriction, indicating that there is no free parameter for these samples. Therefore, the resulting penalty is $\textrm{PT}_{1} = 0.50 \times 1 + 0.25 \times 2 = 1$. For $H_{2}: \theta _{1} > \theta _{2}$, half of the samples are not in agreement with $\theta _{1} > \theta _{2}$ corresponding to one free parameter (i.e., $\textrm{LP}_{1} = 0.50$), while half of the samples are in accordance with $\theta _{1} > \theta _{2}$ corresponding to two free parameters (i.e., $\textrm{LP}_{2} = 0.50$). The resulting penalty is $\textrm{PT}_{2} = 0.50 \times 1 + 0.50 \times 2 = 1.50$. The samples are always in accordance with the unconstrained hypothesis $H_{u}: \theta _{1}, \theta _{2}$ (i.e., $\textrm{LP}_{1} = 0$ and $\textrm{LP}_{2} = 1$). Therefore, the penalty for $H_{u}$ is $\textrm{PT}_{u} = 0 \times 1 + 1 \times 2 = 2$. These hypotheses (containing the $\theta$s defined in the real plane) are called closed convex cones for which the feasible region is infinitely large (denoted by the dark green areas in Fig. 1). Therefore, the penalty is independent of the inspected space (denoted by the circles in Fig. 1). This can be seen by changing the scaling of the circles by multiplying the covariance matrix of MLEs, $\varvec{\hat{\Sigma }}_{\varvec{\hat{\theta }}}$, with the scale factor $c > 0$. Regardless of the size of the inspected space determined by the value of the scale factor *c*, a quarter of the samples are in agreement with $H_{1}: \theta _{1}> 0, \theta _{2} > 0$ and half of the samples are in accordance with $H_{2}: \theta _{1} > \theta _{2}$. Therefore, the proportion of green areas in circles does not depend on the choice of the scale factor *c*. Consequently, the penalties are invariant with respect to the scaling of the covariance matrix, namely, the value of the scale factor *c*.

**Fig. 2:**
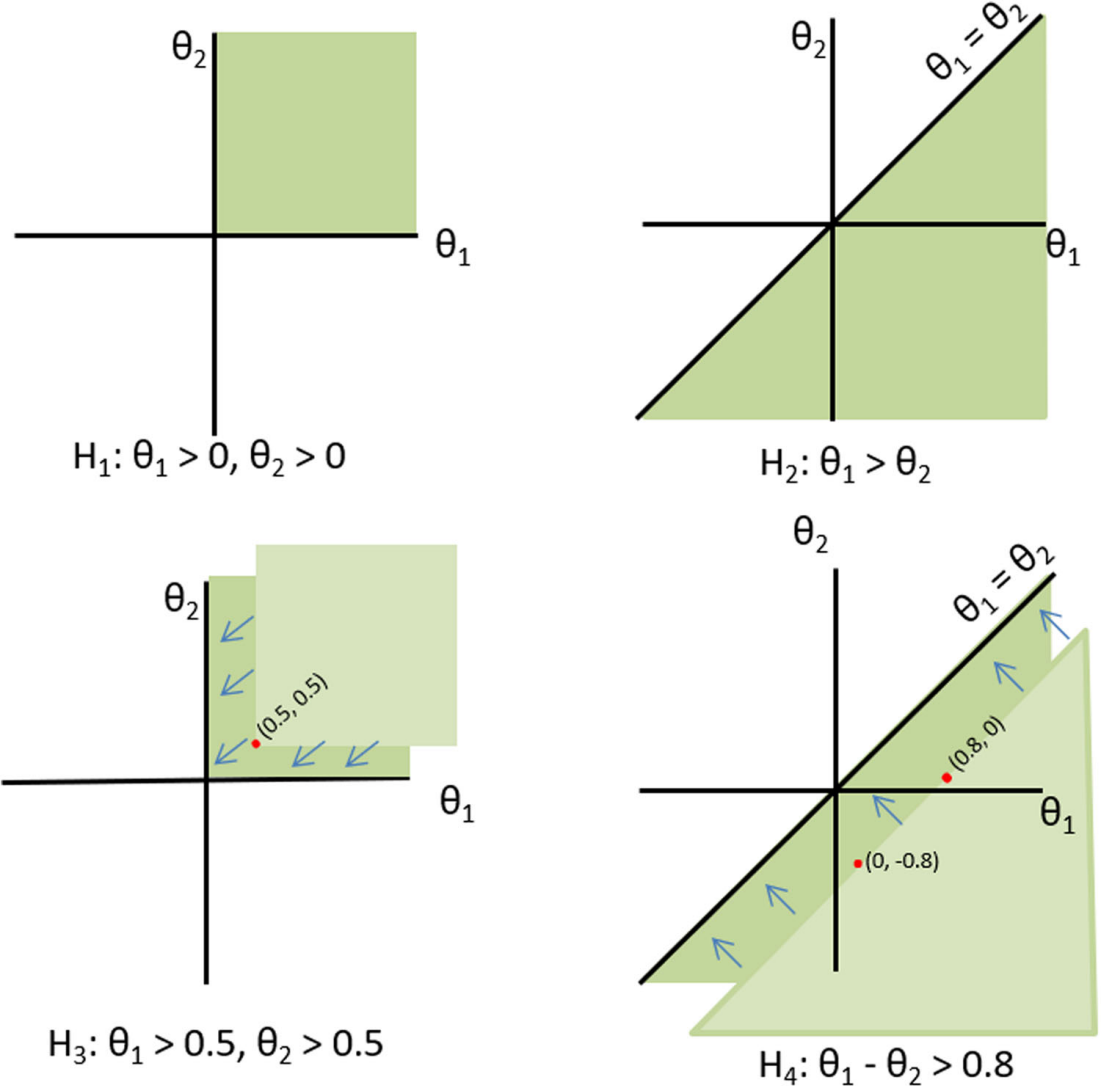
The feasible region (in *green*) for the restrictions of (relocated) closed convex cones

Many hypotheses containing linear restrictions on model parameters (e.g., hypotheses $H_{1}$ and $H_{2}$ in Fig. 1) are of the type closed convex cones (Silvapulle and Sen, 2005, p.82). When hypothesis $H_{m}$ is of the type closed convex cones, the feasible region covered by the restrictions starts from the origin, that is, the point where all elements in $\varvec{\theta }$ are 0 (see the upper panels in Fig. 2). In this case, the mean vector of zeros, $\varvec{\mu }= 0$, is a natural choice. When the feasible region covered by the restrictions does *not* start from the origin, the hypotheses are called relocated closed convex cones (Kuiper et al., 2012, p.2460). For example, hypotheses $H_{3}: \theta _{1}> 0.5, \theta _{2} > 0.5$ and $H_{4}: \theta _{1} - \theta _{2} > 0.8$ are relocated convex cones because their feasible regions do not start from the origin, but become closed convex cones when shifted to the origin. Therefore, we relocate the hypothesis (instead of adjusting $\varvec{\mu }= 0$) such that the region starts from the origin (see the bottom panels in Fig. 2). Since these hypotheses are relocated to the origin, their penalties do not depend on the scaling of the covariance matrix with $c > 0$. This can also be generalized to hypotheses of the forms $H_{m}: \theta _{1}> r_{1}, \theta _{2} > r_{2}$ and $H_{m}: \theta _{1} - \theta _{2} > r_{3}$, where $r_{1}$, $r_{2}$, and $r_{3}$ are constants.

**Fig. 3:**
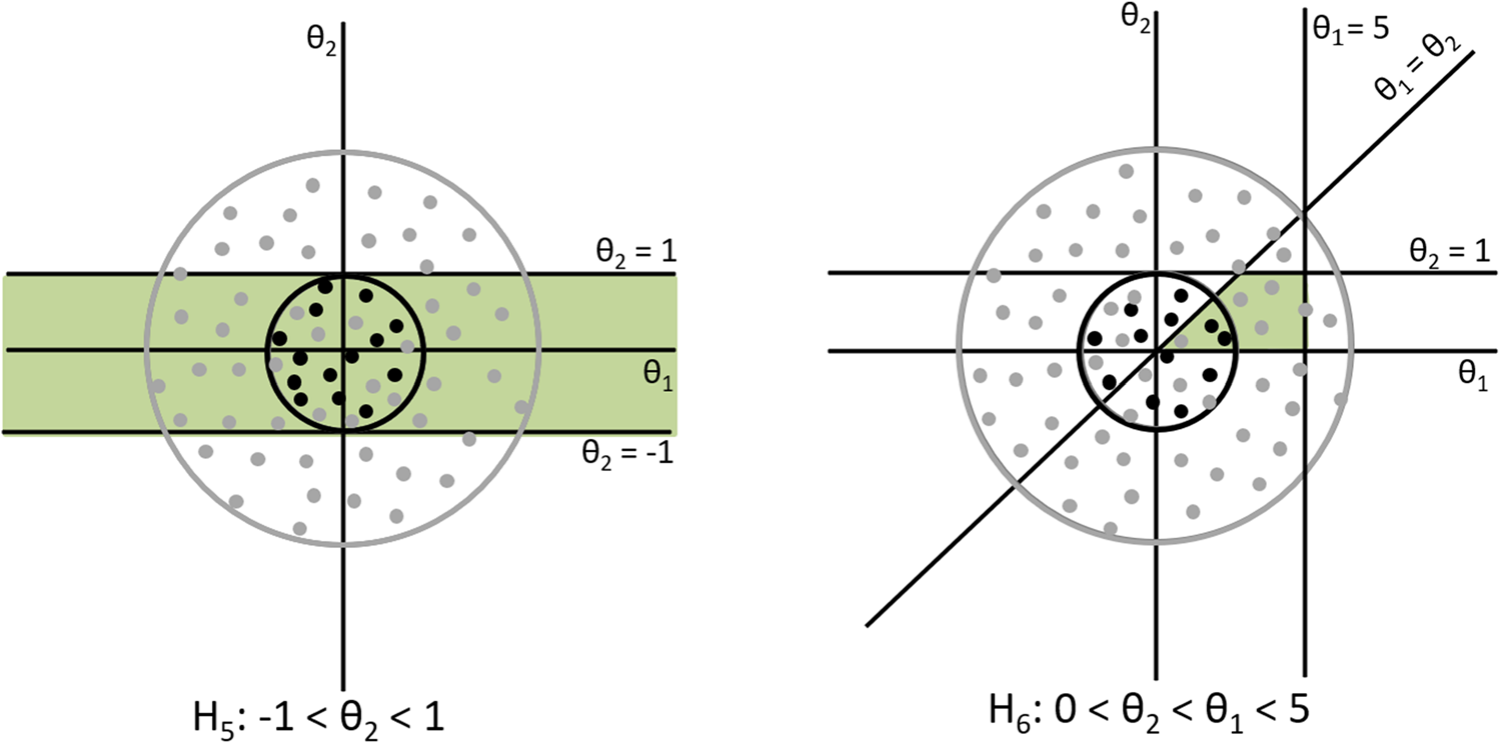
Penalty calculation for non (relocated) closed convex cones with different sizes of inspected spaces (denoted by *circles*)

**Table 2:** Three classes of restrictions for (at least) two-way contingency tables which are exemplified using a $2 \times 4$ contingency table and the corresponding reparameterization into $\eta$ such that they are in accordance with $H_{m}: \varvec{S}_{m}\varvec{\eta } = \varvec{s}_{m}, \varvec{R}_{m}\varvec{\eta } > \varvec{r}_{m}$

	Restrictions on non-marginal	$H_{1}: \pi _{11} - \pi _{21} > \pi _{14} - \pi _{24}$	$\varvec{\eta } = \varvec{\pi } = (\pi _{11}, ..., \pi _{IJ})^{\top }$
Linear restrictions	Cell probabilities	$H_{2}$: $\pi _{21}> \pi _{22}> \pi _{23}> \pi _{24}$	
	Restrictions on marginal	$H_{1}: \pi _{+1} > \{\pi _{+2}, \pi _{+3}, \pi _{+4}\}$	$\varvec{\eta }_{1} = (\pi _{+1}, ..., \pi _{+J})^{\top }$
	Cell probabilities	$H_{2}: \pi _{1+} > \pi _{2+}$	$\varvec{\eta }_{2} = (\pi _{1+}, ..., \pi _{I+})^{\top }$
	Restrictions on conditional	$H_{1}: \frac{\pi _{21}}{\pi _{+1}} > \{\frac{\pi _{22}}{\pi _{+2}}, \frac{\pi _{23}}{\pi _{+3}}, \frac{\pi _{24}}{\pi _{+4}}\}$	$\varvec{\eta }_{1} = (\frac{\pi _{i1}}{\pi _{+1}}, ..., \frac{\pi _{iJ}}{\pi _{+J}})^{\top }$
Non-linear restrictions	Cell probabilities	$H_{2}: \frac{\pi _{12}}{\pi _{1+}} > \frac{\pi _{22}}{\pi _{2+}}$	$\varvec{\eta }_{2} = (\frac{\pi _{1j}}{\pi _{1+}}, ..., \frac{\pi _{Ij}}{\pi _{I+}})^{\top }$
	Restrictions on Local Odds Ratios	$H_{1}: \frac{\pi _{11}\pi _{22}}{\pi _{12}\pi _{21}} = 1, \frac{\pi _{13}\pi _{24}}{\pi _{14}\pi _{23}} > 1$	$\varvec{\eta } = (\frac{\pi _{11}\pi _{22}}{\pi _{12}\pi _{21}}, ..., \frac{\pi _{I-1,J-1}\pi _{I,J}}{\pi _{I-1,J}\pi _{I, J-1}} )^{\top }$
		$$H_{2}: \frac{\pi _{12}\pi _{23}}{\pi _{13}\pi _{22}}> \frac{\pi _{13}\pi _{24}}{\pi _{14}\pi _{23}} > 1$$	

*Note.* $\varvec{\eta }:$ The vector of cell probabilities for linear restrictions of type $H_{m}: \varvec{S}_{m} \varvec{\eta } = \varvec{s}_{m}, \varvec{R}_{m} \varvec{\eta } > \varvec{r}_{m}$ for $i = 1, ..., I$ and $j = 1, ..., J$ or the vector of local odds ratios for $i = 1, ..., I-1$ and $j = 1, ..., J-1$. $\varvec{\eta }_{1}$, $\varvec{\eta }_{2}:$ The vectors of row and column marginal cell probabilities or row and column conditional cell probabilities for $i = 1, ..., I$ and $j = 1, ..., J$, representing the parameters of four different subclasses. $\pi _{i+},$ $\pi _{+j}:$ The cell probabilities summed over the *i*th row and the *j*th column, respectively. For example, $\pi _{1+} = \pi _{11}+\pi _{12}+\pi _{13}+\pi _{14}$ and $\pi _{+1} = \pi _{11} +\pi _{21}$ for a $2 \times 4$ contingency table

In case a hypothesis is *not* of the type (relocated) closed convex cones, LPs, and thus, the penalty, depends on the scaling of the covariance matrix, that is, the choice of *c*. Hypothesis $H_{5}: -1< \theta _{2} < 1$ (see the left panel in Fig. 3) contains range restrictions which are a special case of non (relocated) closed convex cones. For this hypothesis, the proportion of green area in the smaller circle is 1, while for the larger circle it is clearly less than 1. Similarly, for hypothesis $H_{6}: 0< \theta _{2}< \theta _{1} < 5$ (see the right panel in Fig. 3), the proportion of green area decreases as the circle becomes larger. For range restrictions, the penalty depends not only on the choice of *c*, but also on the choice of mean vector $\varvec{\mu }$. In the case of *non* (relocated) closed convex cones, the default penalty calculation behaves as if the scaling parameter *c* is set to infinity. Then, the feasible region will become infinitely small compared to an infinitely large inspected space. We will revisit this topic later in this paper since the cell probabilities always involve bound restrictions, as a special type of range restrictions, which are bounded between 0 and 1 (i.e., $0< \varvec{\pi }< 1$).

**Table 3:** Two other classes of restrictions for (at least) three-way contingency tables which are exemplified using a $4 \times 4 \times 4$ contingency table and the corresponding reparameterization into $\eta$ such that they are in accordance with $H_{m}: \varvec{S}_{m}\varvec{\eta } = \varvec{s}_{m}, \varvec{R}_{m}\varvec{\eta } > \varvec{r}_{m}$

	Classes of restrictions	Examples	Reparameterization into $\eta$s
Non-linear restrictions	Restrictions on marginal	$H_{1}: \frac{\pi _{+11}\pi _{+22}}{\pi _{+12}\pi _{+21}} = \frac{\pi _{+22}\pi _{+33}}{\pi _{+23}\pi _{+32}} = \frac{\pi _{+33}\pi _{+44}}{\pi _{+34}\pi _{+43}}$	$\varvec{\eta }_{1} = (\frac{\pi _{+11}\pi _{+22}}{\pi _{+12}\pi _{+21}}, ..., \frac{\pi _{+J-1,V-1}\pi _{+JV}}{\pi _{+J-1,V}\pi _{+J,V-1}})^{\top }$
	Odds ratios	$H_{2}: \frac{\pi _{1+1}\pi _{2+2}}{\pi _{1+2}\pi _{2+1}}> \frac{\pi _{2+2}\pi _{3+3}}{\pi _{2+3}\pi _{3+2}} > \frac{\pi _{3+3}\pi _{4+4}}{\pi _{3+4}\pi _{4+3}}$	$\varvec{\eta }_{2} = (\frac{\pi _{1+1}\pi _{2+2}}{\pi _{1+2}\pi _{2+1}}, ..., \frac{\pi _{I-1,+,V-1}\pi _{I+V}}{\pi _{I-1,+V}\pi _{I+,V-1}})^{\top }$
		$H_{3}:\frac{\pi _{11+}\pi _{22+}}{\pi _{12+}\pi _{21+}} > \{\frac{\pi _{22+}\pi _{33+}}{\pi _{23+}\pi _{32+}} , \frac{\pi _{33+}\pi _{44+}}{\pi _{34+}\pi _{43+}} \}$	$\varvec{\eta }_{3} = (\frac{\pi _{11+}\pi _{22+}}{\pi _{12+}\pi _{21+}}, ..., \frac{\pi _{I-1,J-1,+}\pi _{IJ+}}{\pi _{I-1,J+}\pi _{I,J-1,+}})^{\top }$
	Restrictions on conditional	$H_{1}: \frac{\pi _{(1)11}\pi _{(1)22}}{\pi _{(1)12}\pi _{(1)21}} = \frac{\pi _{(1)22}\pi _{(1)33}}{\pi _{(1)23}\pi _{(1)32}} = \frac{\pi _{(1)33}\pi _{(1)44}}{\pi _{(1)34}\pi _{(1)43}}$	$\varvec{\eta }_{1} = (\frac{\pi _{(1)11}\pi _{(1)22}}{\pi _{(1)12}\pi _{(1)21}}, ..., \frac{\pi _{(I-1)J-1,V-1}\pi _{(I-1)JV}}{\pi _{(I-1)J-1,V}\pi _{(I-1)J,V-1}})^{\top }$
	Odds ratios	$H_{2}: \frac{\pi _{1(1)1}\pi _{2(1)2}}{\pi _{1(1)2}\pi _{2(1)1}}> \frac{\pi _{2(1)2}\pi _{3(1)3}}{\pi _{2(1)3}\pi _{3(1)2}} > \frac{\pi _{3(1)3}\pi _{4(1)4}}{\pi _{3(1)4}\pi _{4(1)3}}$	$\varvec{\eta }_{2} = (\frac{\pi _{1(1)1}\pi _{2(1)2}}{\pi _{1(1)2}\pi _{2(1)1}}, ..., \frac{\pi _{I-1(J-1)V-1}\pi _{I(J-1)V}}{\pi _{I-1(J-1)V}\pi _{I(J-1)V-1}})^{\top }$
		$H_{3}:\frac{\pi _{11(1)}\pi _{22(1)}}{\pi _{12(1)}\pi _{21(1)}} > \{\frac{\pi _{22(1)}\pi _{33(1)}}{\pi _{23(1)}\pi _{32(1)}} , \frac{\pi _{33(1)}\pi _{44(1)}}{\pi _{34(1)}\pi _{43(1)}} \}$	$\varvec{\eta }_{3} = (\frac{\pi _{11(1)}\pi _{22(1)}}{\pi _{12(1)}\pi _{21(1)}}, ..., \frac{\pi _{I-1,J-1(V-1)}\pi _{IJ(V-1)}}{\pi _{I-1,J(V-1)}\pi _{I,J-1(V-1)}})^{\top }$

*Note.* $\varvec{\eta }_{1}$, $\varvec{\eta }_{2},$ $\varvec{\eta }_{3}:$ The vectors of marginal or conditional odds ratios summed over the first, second, and third variables for $i = 1, ..., I-1$, $j = 1, ..., J-1$, and $v = 1, ..., V-1$, respectively, representing the parameters of six different subclasses. $\pi _{+jv}$, $\pi _{i+v}$, $\pi _{ij+}:$ The cell probabilities summed over one variable. For example, $\pi _{+11} = \pi _{111}+\pi _{211}+\pi _{311}+\pi _{411}$; $\pi _{2+2} = \pi _{212}+\pi _{222}+\pi _{232}+\pi _{242}$; and $\pi _{33+} = \pi _{331}+\pi _{332} + \pi _{333}+\pi _{334}$ for a $4 \times 4 \times 4$ contingency table. $\pi _{(i)jv}$, $\pi _{i(j)v}$, $\pi _{ij(v)}$: The cell probabilities where one level of the corresponding variable is fixed. For example, the subscript (1) in the $\frac{\pi _{1(1)1} \pi _{2(1)2}}{\pi _{1(1)2} \pi _{2(1)1}}$ states that our focus is on the first level of the second variable, and therefore, this level is fixed

In the next section, we elaborate on the classes of restrictions relevant for contingency tables, which are often non-linear in terms of cell probabilities. Hereafter, we elaborate on two main problems for these classes of restrictions and their solutions when using GORICA.

## Classes of restrictions relevant for contingency tables

Let $\varvec{\pi }= (\pi _{111}, ..., \pi _{ijv}, ..., \pi _{IJV})$ denote the cell probabilities in a three-way contingency table, without loss of generalization to other dimensionalities of contingency tables. The parameter $\pi _{ijv}$ is the cell probability for the *i*th level of the first variable, the *j*th level of the second variable, and the *v*th level of the third variable in the contingency table with $i = 1, 2, ..., I$, $j = 1, 2, ..., J$, and $v = 1, 2, ..., V$. As shown by the first two columns in Table 2 for a two-way contingency table and Table 3 for a three-way contingency table, this paper considers five classes of restrictions in the context of contingency tables: Linear restrictions on marginal or non-marginal cell probabilities, restrictions on conditional cell probabilities, restrictions on local odds ratios, restrictions on marginal odds ratios, and restrictions on conditional odds ratios. Notably, the classes of restrictions on local, marginal, and conditional odds ratios can be used to explore the independence between two or three variables in contingency tables using equality restrictions. In line with Agresti (2007, p. 36), the independence between two variables in a two-way contingency table can be investigated by evaluating hypothesis $H_{1}: \frac{\pi _{i-1, j-1}\pi _{ij}}{\pi _{i-1, j}\pi _{i, j-1}} = 1$ (for all *i* and *j*) against its complement in terms of local odds ratios (see Table 2). Similarly, in three-way contingency tables, marginal and conditional independence between variables can be explored in terms of marginal and conditional odds ratios, respectively (see Table 3). Although joint independence is not examined in this study due to the implication of equality restrictions on variables, GORICA can be utilized to evaluate this type of relationship.

The second column in Tables 2 and 3 gives examples of the hypotheses for each subclass of the five classes of restrictions. For instance, the class of restrictions on conditional cell probabilities contains two subclasses for row and column conditional cell probabilities. We anticipate that hypotheses are compared to each other within (but *not* across) these subclasses. Consider hypothesis $H_{1}: \frac{\pi _{21}}{\pi _{+1}} > \{\frac{\pi _{22}}{\pi _{+2}},\frac{\pi _{23}}{\pi _{+3}}, \frac{\pi _{24}}{\pi _{+4}}\}$ in (1) for the gender by earned degrees example presented in the introduction (see “Restrictions on conditional cell probabilities” in Table 2). This hypothesis specifies row-conditional cell probabilities in which the denominators consist of the summations over the levels of the first variable, that is, the rows in the two-way contingency table. The hypothesis states that the proportion of males among bachelor’s degree holders is higher than that among holders of any other degree. Note that hypothesis $H_{1}$ implies that the proportion of females in bachelor’s degree holders is smaller than that in any other degree holders because the male proportion in any degree is one minus the female proportion in the same degree. Hypothesis $H_{1}^{*}: \frac{\pi _{11}}{\pi _{+1}} < \{\frac{\pi _{12}}{\pi _{+2}},\frac{\pi _{13}}{\pi _{+3}}, \frac{\pi _{14}}{\pi _{+4}}\}$ reflects the same restrictions in hypothesis $H_{1}$. Hence, we focus on only one row and formulate the hypothesis in terms of male proportions. $H_{2}: \frac{\pi _{12}}{\pi _{1+}} > \frac{\pi _{22}}{\pi _{2+}}$ is an example of a hypothesis containing column conditional cell probabilities in the same context (see “Restrictions on conditional cell probabilities” in Table 2). It states that the proportion of master’s degree holders for females is larger than that for males. Note that each hypothesis addresses a different research question, namely, the former hypothesis focuses on comparing the probabilities of obtaining degrees by males, and the emphasis in the latter hypothesis is the difference between males and females in obtaining a master’s degree. Hence, it is not reasonable to compare them, that is, they are not competing hypotheses. Consequently, non-competing hypotheses should not be included in the same set, as they should be evaluated separately against their own competing hypotheses.

In Table 2, the first class consists of linear restrictions on the cell probabilities $\varvec{\pi }$, while the other classes contain non-linear restrictions on $\varvec{\pi }$. In Table 3, restrictions on marginal and conditional odds ratios consist of non-linear restrictions on $\varvec{\pi }$. Hypotheses containing non-linear restrictions on $\varvec{\pi }$ cannot (yet) be evaluated with GORICA.

Two main problems have to be solved to apply GORICA in the context of contingency tables: the evaluation of hypotheses containing non-linear restrictions on cell probabilities (Problem A) and the evaluation of hypotheses in contingency tables with empty cells (Problem B). In the following sections, we elaborate on these problems in detail and provide solutions for both of them.

## Problem A: Evaluating non-linear restrictions

GORICA (Altinisik et al., 2021) utilizes the quadratic non-linear optimization method in the solve.QP subroutine of the quadprog package (Turlach, 2014, pp. 2–4), which can only be used to evaluate hypotheses containing linear restrictions on cell probabilities. However, the hypotheses in the context of contingency tables often contain non-linear restrictions on cell probabilities. Another non-linear optimization method can be used to evaluate hypotheses containing non-linear restrictions on cell probabilities. To do this, we use the non-linear optimization method in the auglag subroutine of the alabama package (Varadhan, 2015, pp. 2–5). It should be noted that the evaluation of non-linear restrictions is more challenging than that of linear restrictions. The cell probabilities are always between 0 and 1 and sum to one. Therefore, calculating the penalty in the presence of non-linear restrictions on cell probabilities could be accomplished by directly integrating the bound restrictions $0< \varvec{\pi }< 1$ in the hypothesis of interest. We do not include the bound restrictions in the hypothesis of interest because sampling with bound restrictions is a cumbersome task in our context, see the section “Sampling with or without bound restrictions” in the Online Supplementary Material.

**Fig. 4:**
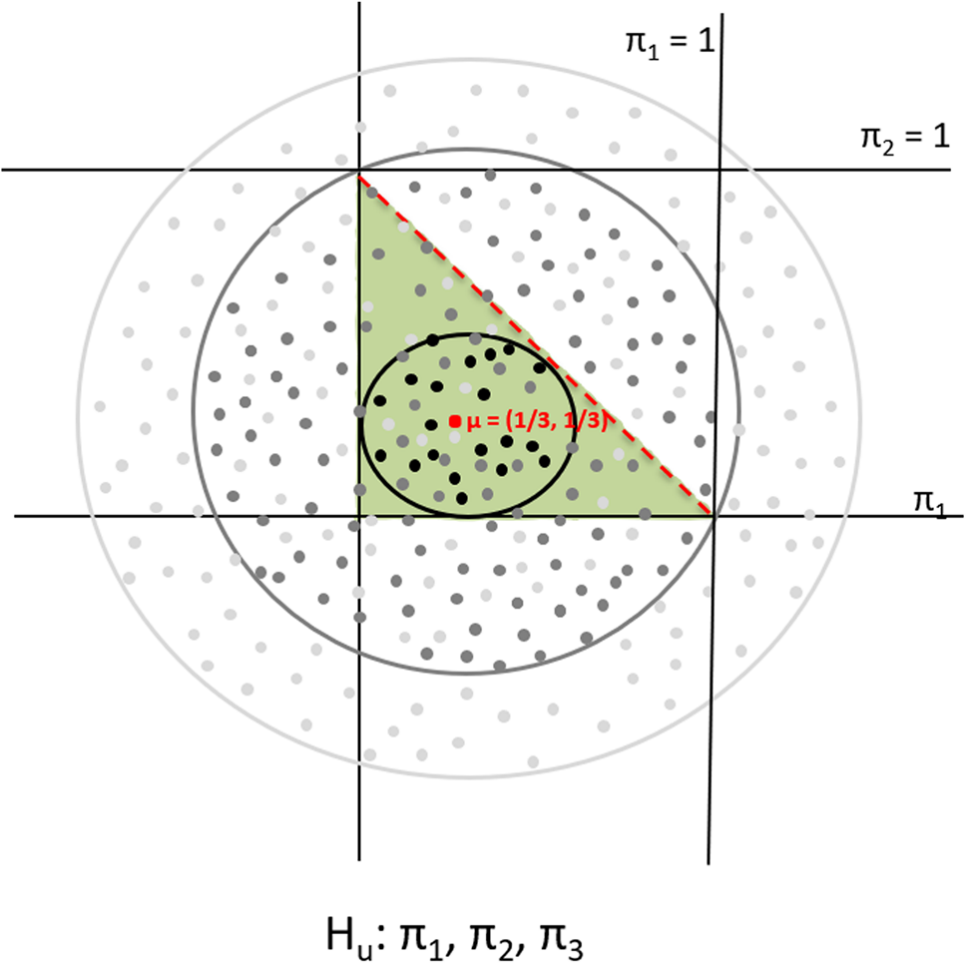
The regular penalty calculation for a bounded unconstrained hypothesis using non-linear optimization. In the figure, $\varvec{\mu }=$ $(\mu _{\pi _{1}}, \mu _{\pi _{2}})$ $= (1/3, 1/3)$, and thus, $\mu _{\pi _{3}} = 1/3$. This is a feasible choice since it assumes that each of the three cells in the contingency table has an equal probability of being observed. The *dashed red line* represents the cases where $\pi _{3} = 0$, and thus, $\pi _{1} + \pi _{2} = 1$. The origin indicates that $\pi _{1} = \pi _{2} = 0$, and thus, $\pi _{3} = 1$

When using the non-linear optimization with bound restrictions, all constraints on $\pi$s must be specified. That necessitates additional bound restrictions such that cell probabilities in the contingency table sum to 1 and each lies between 0 and 1. Notably, the restrictions also hold for the unconstrained hypothesis $H_{u}$ (see Fig. 4), that is, the hypothesis that has no other restrictions on $\pi$s, which acts as a failsafe. The bounded unconstrained hypothesis $H_{u}: \pi _{1}, \pi _{2}, \pi _{3}$ is *not* of the type (relocated) closed convex cones. Therefore, the penalty for the bounded unconstrained hypothesis $H_{u}$ (and all the other hypotheses in $\pi$s) depends on the choices of both the mean vector $\varvec{\mu }$ and the scale factor *c*. If the inspected space (denoted by the circles in Fig. 4) exceeds the boundaries of the feasible region (denoted by the green area in Fig. 4), the proportion of the feasible region to the inspected space reduces. Stated otherwise, the proportion of the green area to the circle in Fig. 4 can become smaller when inspecting a larger circle. The dependence on $\varvec{\mu }$ and *c* in non-linear optimization is shown in more detail in the following subsection. As mentioned, the penalty is invariant with the mean vector $\varvec{\mu }$ and the scale factor *c* when hypotheses contain linear restrictions on cell probabilities. This suggests a solution to the problem that the penalty is not invariant for hypotheses containing non-linear restrictions on cell probabilities: reparameterizing the cell probabilities such that the restrictions become linear in the new parameters.

Let us denote the function of $\varvec{\pi }$ by $\varvec{\eta }= g(\varvec{\pi })$, where the function g(.) differs per subclass as can be seen in the third column of Tables 2 and 3. Note that all subclasses that are non-linear in $\varvec{\pi }$ correspond to hypotheses that are linear in $\varvec{\eta }$. Thus, all hypotheses in Tables 2 and 3 can be expressed in terms of $\varvec{\eta }$as:

4 $$\begin{aligned} H_{m}: \varvec{S}_{m}\varvec{\eta } = \varvec{s}_{m}, \varvec{R}_{m}\varvec{\eta }> \varvec{r}_{m}, \end{aligned}$$

where $\varvec{S}_{m}\in \mathrm I\!R^{h_{s} \times K}$ and $\varvec{R}_{m}\in \mathrm I\!R^{h_{r} \times K}$ are the restriction matrices representing the $h_{s}$ equality and $h_{r}$ inequality constraints in $H_{m}$, respectively, and $\varvec{s}_{m}$ is a $h_{s}$-vector and $\varvec{r}_{m}$ is a $h_{r}$-vector consisting of the constants in $H_{m}$. After reparameterizing cell probabilities, the hypotheses containing linear restrictions on the $\eta$s are evaluated using the quadratic non-linear optimization method (Turlach, 2014, pp. 2–4).

### Dependence of the penalty on $\mu$ and *c* in non-linear optimization

The penalty for hypotheses containing non-linear restrictions on cell probabilities depends on both the mean vector $\varvec{\mu }$ and the scale factor *c*. To illustrate, consider evaluating the bounded hypothesis $H_{1}: \frac{\pi _{21}}{\pi _{+1}} > \{\frac{\pi _{22}}{\pi _{+2}},\frac{\pi _{23}}{\pi _{+3}},\frac{\pi _{24}}{\pi _{+4}}\}$ in (1) against the bounded unconstrained hypothesis $H_{u}: \pi _{11}, \pi _{12}, ..., \pi _{24}$ using the non-linear optimization method for the non-linear restrictions on cell probabilities (Varadhan, 2015, pp. 2–5). Since the contingency table contains $D = 2 \times 4 = 8$ cell probabilities, we adjust the mean vector to the center of the feasible region using $\mu _{i} = 1/D = 0.125$ ($i = 1, 2, ..., 8$), assuming that each cell has equal probability of being observed. Note the linear dependency between cell probabilities, as each of the cell probabilities is one minus the sum of the other cell probabilities. Therefore, the number of free cell probabilities in the contingency table is $D - 1 = 7.000$. In Table 4, the penalties for $H_{1}$ and $H_{u}$ (both containing bound restrictions) are given for $c = 1$ and $c = 100000$. The penalty for hypothesis $H_{1}$ depends on *c*, namely, $PT_{1}(\varvec{\pi })$ = 6.322 for $c = 1$, while $PT_{1}(\varvec{\pi })$ = 0.884 for $c = 100000$. For $c = 1$, the circle is contained in the feasible space of the unconstrained hypothesis $H_{u}$. Therefore, the penalty for the unconstrained hypothesis $H_{u}$ is equal to the expected number of free parameters, namely, $PT_{u}(\varvec{\pi }) = D - 1 = 7.000$, see the smallest circle in Fig. 4. Similarly, the penalty for the bounded unconstrained hypothesis $H_{u}$ varies with *c*, namely, $PT_{u}(\varvec{\pi }) = 7.000$ for $c = 1$, while $PT_{u}(\varvec{\pi }) = 2.328$ for $c = 100000$.

**Table 4:** Penalty calculation for hypotheses in terms of $\pi$s based on the covariance matrix $c\varvec{\hat{\Sigma }}_{\varvec{\hat{\pi }}}$

	Penalty term	
Hypothesis with		
bound restrictions	$c=1$	$c=100000$
$$ H_{1}$$: $$\frac{\pi _{21}}{\pi _{+1}} > \{\frac{\pi _{22}}{\pi _{+2}},\frac{\pi _{23}}{\pi _{+3}},\frac{\pi _{24}}{\pi _{+4}}\}$$	6.322	0.884
$H_{u}$: $\pi _{11}, \pi _{12}, ..., \pi _{24}$	7.000	2.328

### Reparameterizing cell probabilities

After reparameterizing cell probabilities, the penalty is invariant to *c* for each hypothesis under evaluation. Hypothesis $H_{1}: \frac{\pi _{21}}{\pi _{+1}} > \{\frac{\pi _{22}}{\pi _{+2}},\frac{\pi _{23}}{\pi _{+3}}, \frac{\pi _{24}}{\pi _{+4}}\}$ in (1) can be rewritten as $H_{1}: \eta _{1} > \{\eta _{2}, \eta _{3}, \eta _{4}\}$, which is linear in $\varvec{\eta }$, with $\varvec{\eta }= (\eta _{1},\eta _{2},\eta _{3},\eta _{4})^{\top } = (\frac{\pi _{21}}{\pi _{+1}}, \frac{\pi _{22}}{\pi _{+2}}, \frac{\pi _{23}}{\pi _{+3}}, \frac{\pi _{24}}{\pi _{+4}})^{\top }$ and

$$\varvec{R}_{1}= \left( \begin{array}{cccc} 1 & -1 & 0 & 0 \\ 1 & 0 & -1 & 0 \\ 1 & 0 & 0 & -1 \\ \end{array} \right) , \varvec{r}_{1}= \left( \begin{array}{c} 0 \\ 0 \\ 0 \\ \end{array} \right) .$$

As is to be expected for hypotheses of the form in (4), the penalty for hypothesis $H_{1}: \eta _{1} > \{\eta _{2}, \eta _{3}, \eta _{4}\}$ is independent of *c*, that is, $PT_{1}(\varvec{\eta })$ = 2.547 for $c = 1$ and $c =$ 100000 (see Table 5). The penalty for the unconstrained hypothesis $H_{u}$ is now based on the number of $\eta$ parameters, that is, $H_{u}: \eta _{1}, \eta _{2}, \eta _{3}, \eta _{4}$, and, therefore, $PT_{u}(\varvec{\eta }) = 4.000$ for $c = 1$ and $c = 100000$. Hence, by reparameterizing cell probabilities in terms of linear restrictions in the new parameters, the GORICA in (2) can be applied to evaluate hypotheses containing non-linear restrictions on cell probabilities using $\varvec{\theta }= \varvec{\eta }$.

After reparameterization, the bound restrictions may also hold for the new parameters $\varvec{\eta }$. However, these bound restrictions do not exert an influence on the relative differences between penalties because of the same reason explained in the section “Sampling with or without bound restrictions” in the Online Supplementary Material.

**Table 5:** Penalty calculation for hypotheses in terms of $\eta$s based on the covariance matrix $c\varvec{\hat{\Sigma }}_{\varvec{\hat{\eta }}}$

	Penalty term	
Hypothesis with		
unbounded $\eta$s	$c=1$	$c=100000$
$H_{1}$: $\eta _{1} > \{ \eta _{2}, \eta _{3}, \eta _{4} \}$	2.547	2.547
$H_{u}$: $\eta _{1}, \eta _{2}, \eta _{3}, \eta _{4}$	4.000	4.000

### Comparing relative differences in penalties

Reparameterizing cell probabilities raises the argument of whether this affects the relative differences between penalties in the original scale because equivalent hypotheses should have similar relative differences between penalties across different parameterizations. In response to this query, we use the penalty weights (not the pairwise ratios between penalties) which have the same functional characteristic as the Akaike weight (Burnham and Anderson, 2002, p. 75). In line with the Akaike weight, the penalty weight for hypothesis $H_{m}$ is calculated by replacing $\textrm{AIC}_{m}$ by $2\textrm{PT}_{m}$:

5 $$\begin{aligned} {w_{m}^{P}=\frac{\textrm{exp} \{\mathrm {-}\mathrm {\text PT}_{m}\}}{\hspace{-0.2cm}{\sum _{m' = \textrm{1}}^{M}}\textrm{exp}\{\mathrm {-\text PT}_{m'}\}}}, \end{aligned}$$

where *M* is the number of hypotheses in the set, including the unconstrained hypothesis $H_{u}$.

The penalty weights can be used to explore the extent of the effect of *c* on the penalty. The penalty weights of hypothesis $H_{1}: \frac{\pi _{21}}{\pi _{+1}} > \{\frac{\pi _{22}}{\pi _{+2}},\frac{\pi _{23}}{\pi _{+3}},\frac{\pi _{24}}{\pi _{+4}}\}$ (including bound restrictions) against the unconstrained hypothesis $H_{u}: \pi _{11}, \pi _{12}, ..., \pi _{24}$ differ for $c = 1$ and $c = 100000$. That is, $w_{1}^{P} = \frac{\textrm{exp}(-6.322)}{\textrm{exp}(-6.322) + \textrm{exp}(-7.000)} = 0.663$ for $c = 1$ and $w_{1}^{P} = \frac{\textrm{exp}(-0.884)}{\textrm{exp}(-0.884) + \textrm{exp}(-2.328)} = 0.809$ for $c = 100000$ (see the first line in Table 6). Consequently, the resulting penalty weights of the unconstrained hypothesis $H_{u}$ in the set with hypothesis $H_{1}$ are also not close to each other, that is, $w_{u}^{P} = 1 - 0.663 = 0.337$ for $c = 1$ and $w_{u}^{P} = 1 - 0.809 = 0.191$ for $c = 100000$ (see the second line in Table 6). In contrast, after reparameterizing cell probabilities in terms of $\varvec{\eta }$, the penalty weight of hypothesis $H_{1}: \eta _{1} > \{\eta _{2}, \eta _{3}, \eta _{4} \}$ against the unconstrained hypothesis $H_{u}: \eta _{1}, \eta _{2}, \eta _{3}, \eta _{4}$ are identical for $c = 1$ and $c = 100000$. That is, $w_{1}^{P} = \frac{\textrm{exp}(-2.547)}{\textrm{exp}(-2.547) + \textrm{exp}(-4.000)} = 0.810$ for $c = 1$ and $c = 100000$ (see the third line in Table 6). Similarly, $w_{u}^{P} = \frac{\textrm{exp}(-4.000)}{\textrm{exp}(-2.547) + \textrm{exp}(-4.000)} = 0.190$ for $c = 1$ and $c = 100000$ (see the fourth line in Table 6). For $c = 100000$, the penalty weights across the non-linear optimizations on $\varvec{\pi }$ and $\varvec{\eta }$ are quite close to each other when evaluating hypothesis $H_{1}$ against the unconstrained hypothesis $H_{u}$ (i.e., $w_{1}^{P}$ $= 0.809$ for the non-linear optimization with including the bound restrictions on $\varvec{\pi }$ and $w_{1}^{P}$ $= 0.810$ for the quadratic non-linear optimization without including the bound restrictions on $\varvec{\eta }$).

**Table 6:** Penalty calculation (weights) for hypotheses in terms of $\pi$s based on $c\varvec{\hat{\Sigma }}_{\varvec{\hat{\pi }}}$ and $\eta$s based on $c\varvec{\hat{\Sigma }}_{\varvec{\hat{\eta }}}$

	$c=1$		$c = 100000$	
	$\mathrm {PT_{m}}$	$w_{m}^{P}$	$\mathrm {PT_{m}}$	$w_{m}^{P}$
Hypothesis with bound restrictions				
$$ H_{1}$$: $$\frac{\pi _{21}}{\pi _{+1}} > \{\frac{\pi _{22}}{\pi _{+2}},\frac{\pi _{23}}{\pi _{+3}},\frac{\pi _{24}}{\pi _{+4}}\}$$	6.322	0.663	0.884	**0.809**
$H_{u}$: $\pi _{11}, \pi _{12}, ..., \pi _{24}$	7.000	0.337	2.328	**0.191**
Hypothesis with unbounded $\eta$s				
$$ H_{1}$$: $$\eta _{1} > \{ \eta _{2}, \eta _{3}, \eta _{4} \}$$	2.547	**0.810**	2.547	**0.810**
$H_{u}$: $\eta _{1}, \eta _{2}, \eta _{3}, \eta _{4}$	4.000	**0.190**	4.000	**0.190**

*Note.* $\varvec{\eta }= (\eta _{1}, \eta _{2}, \eta _{3}, \eta _{4})^{\top } = (\frac{\pi _{21}}{\pi _{+1}}, \frac{\pi _{22}}{\pi _{+2}},\frac{\pi _{23}}{\pi _{+3}},\frac{\pi _{24}}{\pi _{+4}})^{\top }.$ The penalty for $H_{1}$: $\frac{\pi _{21}}{\pi _{+1}} > \{\frac{\pi _{22}}{\pi _{+2}},\frac{\pi _{23}}{\pi _{+3}},\frac{\pi _{24}}{\pi _{+4}}\}$ is calculated using the non-linear optimization the auglag subroutine of the alabama package (Varadhan, 2015, pp. 2–5). The bold text indicates the penalty weights that are close to each other

It should be noted that a similar situation arises for hypotheses containing equality restrictions. To illustrate, consider the evaluation of $H_{2}: \frac{\pi _{21}}{\pi _{+1}} = \frac{\pi _{22}}{\pi _{+2}} = \frac{\pi _{23}}{\pi _{+3}} = \frac{\pi _{24}}{\pi _{+4}}$, which states that the four conditional cell probabilities are equal to each other. If we evaluate hypothesis $H_{2}$ against the unconstrained hypothesis $H_{u}: \pi _{11}, \pi _{12}, ..., \pi _{24}$ using the non-linear optimization with the bound restrictions, the penalty weights of hypothesis $H_{2}$ deviate from each other for $c = 1$ and $c = 100000$. That is, $w_{2}^{P} = \frac{\textrm{exp}(-4.000)}{\textrm{exp}(-4.000) + \textrm{exp}(-7.000)} = 0.953$ for $c = 1$ and $w_{2}^{P} = \frac{\textrm{exp}(-0.443)}{\textrm{exp}(-0.443) + \textrm{exp}(-2.328)} = 0.868$ for $c = 100000$. In contrast, after reparameterizing cell probabilities, if we evaluate hypothesis $H_{2}: \eta _{1} = \eta _{2} = \eta _{3} = \eta _{4}$ against the unconstrained hypothesis $H_{u}: \eta _{1}, \eta _{2}, \eta _{3}, \eta _{4}$ using the quadratic non-linear optimization without the bound restrictions, the same penalty weight is obtained for $c = 1$ and $c = 100000$, that is, $w_{2}^{P} = \frac{\textrm{exp}(-1.000)}{\textrm{exp}(-1.000) + \textrm{exp}(-4.000)} = 0.953$. Note that when hypotheses contain only equality restrictions, both non-linear optimization methods yield identical penalty weights for $c = 1$.

For more details on the comparisons of penalty weights for the hypotheses in Table 2, see the section “Comparing penalty weights between non-linear optimizations” in the Online Supplementary Material. We provide multiple examples to investigate the effect of reparameterizing cell probabilities on the relative differences between penalties. For this purpose, we use the gender by earned degrees data in Table 1 and the hypotheses in Table 2. We use the non-linear optimization method with including the bound restrictions on $\varvec{\pi }$ and the quadratic non-linear optimization method without including the bound restrictions on $\varvec{\eta }$ for $c = 1$ and $c = 100000$. We showed that for either $c = 1$ or $c = 100000$, the quadratic non-linear optimization on $\varvec{\eta }$ yields reasonably close penalty weights compared to the non-linear optimization on $\varvec{\pi }$.

### The advantages of reparameterization

The non-linear optimization, including the bound restrictions on $\varvec{\pi }$ is a time-consuming process, particularly for high-dimensional contingency tables. It is often tedious to use the many cell probabilities in the contingency table to specify the hypotheses of interest. Besides, the last cell probability has to be specified as one minus the sum of the other cell probabilities (due to the linear dependency among them). As can be seen in the supplementary material, the non-linear optimization on $\varvec{\eta }$ provides reasonably close penalty weights with the non-linear optimization on $\varvec{\pi }$ for either $c = 1$ or $c = 100000$. Moreover, regardless of the choice of *c* and the type of restrictions, both optimizations selected the same hypothesis as the best hypothesis in the set. We will also conduct a simulation study after the next section showing that the non-linear optimization on $\varvec{\eta }$ performs quite satisfactorily when evaluating hypotheses containing non-linear restrictions on cell probabilities. Considering all these together, reparameterizing cell probabilities, and consequently, using the non-linear optimization on $\varvec{\eta }$, is an alternative method that is easier to implement using the general solution in the $\texttt {gorica}$ package (Van Lissa et al., 2020).

## Problem B: Empty cells due to sampling zeros

High-dimensional sparse contingency tables often contain empty cells which may complicate hypotheses evaluation with GORICA in (2) using $\varvec{\theta }= \varvec{\eta }$. Empty cells due to zeros in contingency tables may cause some of the estimates of $\eta$s to be 0 or 1, or some of the $\eta$s to be inestimable. There are two types of zeros in the literature that may cause empty cells in contingency tables: sampling zeros and structural zeros. Sampling zeros are observed as zeros in contingency tables because of sampling variability, although they have the potential to be a positive count. Structural zeros are inherently zero without variability and do not have the potential to be observed as a positive count. Although the empty cells due to sampling and structural zeros cause similar problems, they require different solutions when evaluating hypotheses using GORICA. This section elaborates on our solution to deal with empty cells due to sampling zeros. Our solution to empty cells due to structural zeros is elaborated in the section “Hypothesis evaluation in the presence of structural zeros” in the Online Supplementary Material. The solution for structural zeros involves rewriting the hypotheses of interest. We provide brief guidance and a summary table on rewriting hypotheses in the subsection “Guidance on rewriting hypotheses” in the online supplementary material.

Many solutions in the literature can be used to deal with sampling zeros in contingency tables. However, most of these solutions are only ad hoc solutions and/or cannot be used with GORICA. One of the most common solutions in contingency tables is combining the levels of variables. However, this is only an ad hoc solution which may cause information loss (Shields & Heeler, 1979; Agresti, 2007). Moreover, we often cannot specify the hypotheses of interest after collapsing the levels of categorical variables. Because this often distorts the relationship between structural parameters by discarding some of the levels, and thus, structural parameters, from the model. Alternatively, Brzezińska (2015, p. 53) elaborates on six possible solutions (based on a literature search) to deal with sampling zeros in contingency tables. Each of the six solutions preserves the relationship between structural parameters, none of which eliminates structural parameters from the model. One of the solutions involves increasing the sample size to eliminate sampling zeros in contingency tables. However, increasing the sample size is often burdensome, if not impossible, and should be accomplished at the study design phase. Another solution is particularly needed when a function of cell probabilities is inestimable due to zero occurrence both in numerator and denominator. Additionally, four of the solutions involve adding a small constant to each (zero) cell in contingency tables. These solutions may require user intervention involving rewriting the hypotheses of interest. However, adding a small constant to each (zero) cell in contingency tables may not add variability to the estimates of $\eta$s in bootstrap samples. Hence, we propose an alternative solution that is suitable for evaluating theory-based hypotheses in the presence of sampling zeros.

Empty cells in contingency tables may cause some of the $\eta$ estimates to be zero, which can occur in all classes mentioned in Tables 2 and 3. To illustrate this problem, consider evaluating hypothesis $H_{m}: \eta _{1}> \eta _{2} > \eta _{3}$ with $\varvec{\eta }= (\eta _{1}, \eta _{2}, \eta _{3})^\top$ = $(\frac{\pi _{11}}{\pi _{11}+\pi _{21}}, \frac{\pi _{12}}{\pi _{12} + \pi _{22}}, \frac{\pi _{13}}{\pi _{13} + \pi _{23}})^\top$ based on the $2 \times 3$ contingency table containing the true cell probabilities ($\pi _{ij}$) and observed cell frequencies ($y_{ij}$) between brackets:

**Table:** 

$\pi _{11}$ (7)	$\pi _{12}$ (0)	$\pi _{13}$ (6)
$\pi _{21}$ (3)	$\pi _{22}$ (2)	$\pi _{23}$ (3)

The frequency belonging to the first row and second column is observed as zero (i.e., $y_{12} = 0$). Consequently, the MLE of the corresponding cell probability is zero (i.e., $\hat{\pi }_{12} = 0$). Therefore, $\eta _{2} = \frac{\pi _{12}}{\pi _{12} + \pi _{22}}$ is estimated as zero because of an empty cell, that is, $\hat{\eta }_{2} = 0$. Then, the covariance matrix of $\varvec{\hat{\eta }} = (\hat{\eta }_{1}, 0, \hat{\eta }_{3})^{\top }$ is:

$$ \varvec{\hat{\Sigma }}_{\varvec{\hat{\eta }}} = \begin{bmatrix} \begin{array}{*{3}c} \textrm{Var}(\hat{\eta }_{1})& 0 & \textrm{Cov}(\hat{\eta }_{1}, \hat{\eta }_{3}) \\ 0 & 0 & 0 \\ \textrm{Cov}(\hat{\eta }_{1}, \hat{\eta }_{3}) & 0 & \textrm{Var}(\hat{\eta }_{3}) \end{array} \end{bmatrix}. $$

This covariance matrix is not positive definite because it contains only zeros in the second row and column.

Sometimes, some of the $\eta$ parameters can be estimated as one because of empty cells. This happens for the class of restrictions on conditional cell probabilities in Table 2. Consider evaluating hypothesis $H_{m}: \eta _{1} > \eta _{2}$ with $\varvec{\eta } = (\eta _{1}, \eta _{2})^{\top } = (\frac{\pi _{11}}{\pi _{11}+\pi _{21}}, \frac{\pi _{12}}{\pi _{12}+\pi _{22}})^\top$, where $\hat{\pi }_{22} = 0$ based on the $2 \times 2$ contingency table:

**Table:** 

$\pi _{11}$ (7)	$\pi _{12}$ (5)
$\pi _{21}$ (8)	$\pi _{22}$ (0)

This results in $\hat{\eta }_{2} = \frac{\hat{\pi }_{12}}{\hat{\pi }_{12}+\hat{\pi }_{22}} = 1$ which has no variation. Because of the lack of variation, we cannot calculate the order-restricted maximum log-likelihood and penalty when inspecting this hypothesis.

Similarly, sometimes, some of the $\eta$ parameters cannot be estimated because of empty cells. This occurs for the classes of restrictions on conditional cell probabilities in Table 2 and the odds ratios in Tables 2 and 3. An example of this situation is provided by evaluating hypothesis $H_{m}: \eta _{1} > 1$ with $\eta _{1} = \frac{\pi _{11}\pi _{22}}{\pi _{12}\pi _{21}}$, where $\hat{\pi }_{12} = 0$ because of an empty cell in the $2 \times 4$ contingency table:

**Table:** 

$\pi _{11}$ (5)	$\pi _{12}$ (0)	$\pi _{13}$ (15)	$\pi _{14}$ (7)
$\pi _{21}$ (3)	$\pi _{22}$ (8)	$\pi _{23}$ (0)	$\pi _{24}$ (6)

Thus, when inspecting $H_{m}: \eta _{1} > 1$, we cannot calculate the order-restricted maximum log-likelihood and penalty.

### Adjusting the covariance matrix in the presence of sampling zeros

Our solution is simple and does not require user intervention. The solution involves adjusting the covariance matrix $\varvec{\hat{\Sigma }}_{\varvec{\hat{\eta }}}$ to obtain the order-restricted maximum log-likelihood and penalty parts of GORICA. We replace the zeros on the diagonal (which are the variances of sampling zeros based on the data) of $\varvec{\hat{\Sigma }}_{\varvec{\hat{\eta }}}$ with the lowest variance in the same matrix. Suppose that $\varvec{\hat{\eta }} = (\hat{\eta }_{1}, 0, \hat{\eta }_{3})^{\top }$ and $\hat{\eta }_{2} = 0$ because of sampling zero(s). Then, the covariance matrix of $\varvec{\hat{\eta }}$

$$ \varvec{\hat{\Sigma }}_{\varvec{\hat{\eta }}} = \begin{bmatrix} \begin{array}{*{3}c} \textrm{Var}(\hat{\eta }_{1})& 0 & \textrm{Cov}(\hat{\eta }_{1}, \hat{\eta }_{3}) \\ 0 & 0 & 0 \\ \textrm{Cov}(\hat{\eta }_{1}, \hat{\eta }_{3}) & 0 & \textrm{Var}(\hat{\eta }_{3}) \end{array} \end{bmatrix} $$

will be replaced by the adjusted covariance matrix

$$ \varvec{\hat{\Sigma }}^{adj}_{\varvec{\hat{\eta }}} \!=\! \begin{bmatrix} \begin{array}{*{3}c} \textrm{Var}(\hat{\eta }_{1})& 0 & \textrm{Cov}(\hat{\eta }_{1}, \hat{\eta }_{3}) \\ 0 & \!\textrm{min}\{\textrm{Var}(\hat{\eta }_{1}), \textrm{Var}(\hat{\eta }_{3}) \} & 0 \\ \!\!\textrm{Cov}(\hat{\eta }_{1}, \hat{\eta }_{3}) & \!\!0 & \!\!\textrm{Var}(\hat{\eta }_{3}) \end{array} \end{bmatrix}. \!$$

**Table 7:** Contingency tables simulation: The population values of cell probabilities for the four cases

Cramer’s V	$\pi _{11}$	$\pi _{12}$	$\pi _{13}$	$\pi _{14}$	$\pi _{21}$	$\pi _{22}$	$\pi _{23}$	$\pi _{24}$
Case 1: $H_{1}: \eta _{1} > \{\eta _{2}, \eta _{3}, \eta _{4}\}$								
$\Phi = 0.10$	0.1170	0.1170	0.1170	0.1170	0.1810	0.1170	0.1170*	0.1170
$\Phi = 0.30$	0.0787	0.0787	0.0787	0.0787	0.3706	0.0787	0.0787*	0.1573
$\Phi = 0.50$	0.0224	0.0224	0.0224	0.0224	0.7986	0.0224	0.0224*	0.0670
Case 2: $H_{2}: \{\eta _{1} = \eta _{2}\}, \{\eta _{3} > \eta _{4}\}$								
$\Phi = 0.10$	0.0569	0.0569	0.1708	0.1708	0.0569*	0.0569*	0.2598	0.1710
$\Phi = 0.30$	0.0413	0.0413	0.1237	0.1237	0.0413*	0.0413*	0.4636	0.1238
$\Phi = 0.50$	0.0347	0.0347	0.1040	0.1040	0.0347*	0.0347*	0.6185	0.0347
Case 3: $H_{3}: \eta _{1}> \eta _{2}> \eta _{3} > \eta _{4}$								
$\Phi = 0.10$	0.1071	0.1071	0.1071	0.1071	0.1857	0.1501	0.1287	0.1071*
$\Phi = 0.30$	0.0618	0.0618	0.0618	0.0618	0.4069	0.1853	0.0988	0.0618*
$\Phi = 0.50$	0.0075	0.0199	0.0199	0.0199	0.4979	0.2324	0.1992	0.0033*
Case 4: $H_{u}: \eta _{1}, \eta _{2}, \eta _{3}, \eta _{4}$								
$\Phi = 0.10$	0.1070	0.1070	0.1070	0.1070	0.1071*	0.1287	0.1501	0.1861
$\Phi = 0.30$	0.0581	0.0581	0.0581	0.0581	0.0614*	0.0988	0.2015	0.4059
$\Phi = 0.50$	0.0560	0.0514	0.0514	0.0514	0.0017*	0.1028	0.2570	0.4283

*Note.* $\varvec{\eta } = (\eta _{1}, \eta _{2}, \eta _{3}, \eta _{4})^{\top } = (\frac{\pi _{21}}{\pi _{+1}},\frac{\pi _{22}}{\pi _{+2}}, \frac{\pi _{23}}{\pi _{+3}},\frac{\pi _{24}}{\pi _{+4}})^{\top }.$ The asterisk (*) indicates that the observed frequency for the corresponding cell is set to zero, after the data were generated, to include the sampling zeros in the contingency tables

Note that we propose a data-dependent solution and this solution does not guarantee the construction of a positive definite covariance matrix. Replacement of zeros with the smallest variance in the same matrix might be a more reasonable solution when compared to that with a larger value, e.g., $\textrm{mean}\{\textrm{Var}(\hat{\eta }_{1}), \textrm{Var}(\hat{\eta }_{3})\}$ or $\textrm{max}\{\textrm{Var}(\hat{\eta }_{1}), \textrm{Var}(\hat{\eta }_{3})\}$, because the latter solutions incorporate more unfounded information into the covariance matrix when evaluating theory-based hypotheses. A small count often has a smaller variance than a large count across bootstrap samples. In our solution, we tried to make the smallest change possible in the covariance matrix that would be compatible with the data. However, for the same reason, the latter solutions may perform better than our solution in dealing with the non-positive definiteness of the covariance matrix. Therefore, we recommend applying our solution first and then (if a positive definite covariance matrix cannot be obtained) applying functions that produce higher variance values for the zeros on the diagonal, such as mean and max functions.

Our solution above also applies in the cases where some of the $\eta$ parameters are estimated as one or cannot be estimated because of sampling zeros. To investigate how well our solution works, we will conduct a simulation study in the next section.

## Performance of GORICA for contingency tables

We conduct a simulation study for evaluating hypotheses containing non-linear restrictions on cell probabilities in the presence of empty cell(s) due to sampling zero(s). It was mentioned in the introduction that the evaluation of the hypothesis $H_{1}: \frac{\pi _{21}}{\pi _{+1}} > \{\frac{\pi _{22}}{\pi _{+2}},\frac{\pi _{23}}{\pi _{+3}},\frac{\pi _{24}}{\pi _{+4}}\}$ in (1) might be of interest to researchers. Let us assume that the researchers are interested in the evaluation of this hypothesis together with two competing hypotheses, and the unconstrained hypothesis $H_{u}$:

6 $$\begin{aligned} H_{1}:&\frac{\pi _{21}}{\pi _{+1}}> \{\frac{\pi _{22}}{\pi _{+2}},\frac{\pi _{23}}{\pi _{+3}},\frac{\pi _{24}}{\pi _{+4}}\}, \nonumber \\ H_{2}:&\{\frac{\pi _{21}}{\pi _{+1}} = \frac{\pi _{22}}{\pi _{+2}}\}, \{\frac{\pi _{23}}{\pi _{+3}}> \frac{\pi _{24}}{\pi _{+4}}\}, \nonumber \\ H_{3}:&\frac{\pi _{21}}{\pi _{+1}}> \frac{\pi _{22}}{\pi _{+2}}> \frac{\pi _{23}}{\pi _{+3}} > \frac{\pi _{24}}{\pi _{+4}},\\ H_{u}:&\frac{\pi _{21}}{\pi _{+1}}, \frac{\pi _{22}}{\pi _{+2}}, \frac{\pi _{23}}{\pi _{+3}}, \frac{\pi _{24}}{\pi _{+4}}. \nonumber \end{aligned}$$

Hypothesis $H_{2}$ states that the proportions of males in bachelor’s degree holders and master’s degree holders are the same, while the proportion of males in professional degree holders is larger than that in doctorate degree holders. Hypothesis $H_{3}$ specifies that the proportion of males in doctorate degree holders is the smallest followed by that for professional, master’s, and bachelor’s degree holders, respectively. The unconstrained hypothesis $H_{u}$ contains all possible relations among cell probabilities (including the ones in hypotheses $H_{1}$, $H_{2}$, and $H_{3}$) which is used as a failsafe in case $H_{1}$, $H_{2}$, and $H_{3}$ turn out to be weak hypotheses in the set.

**Fig. 5:**
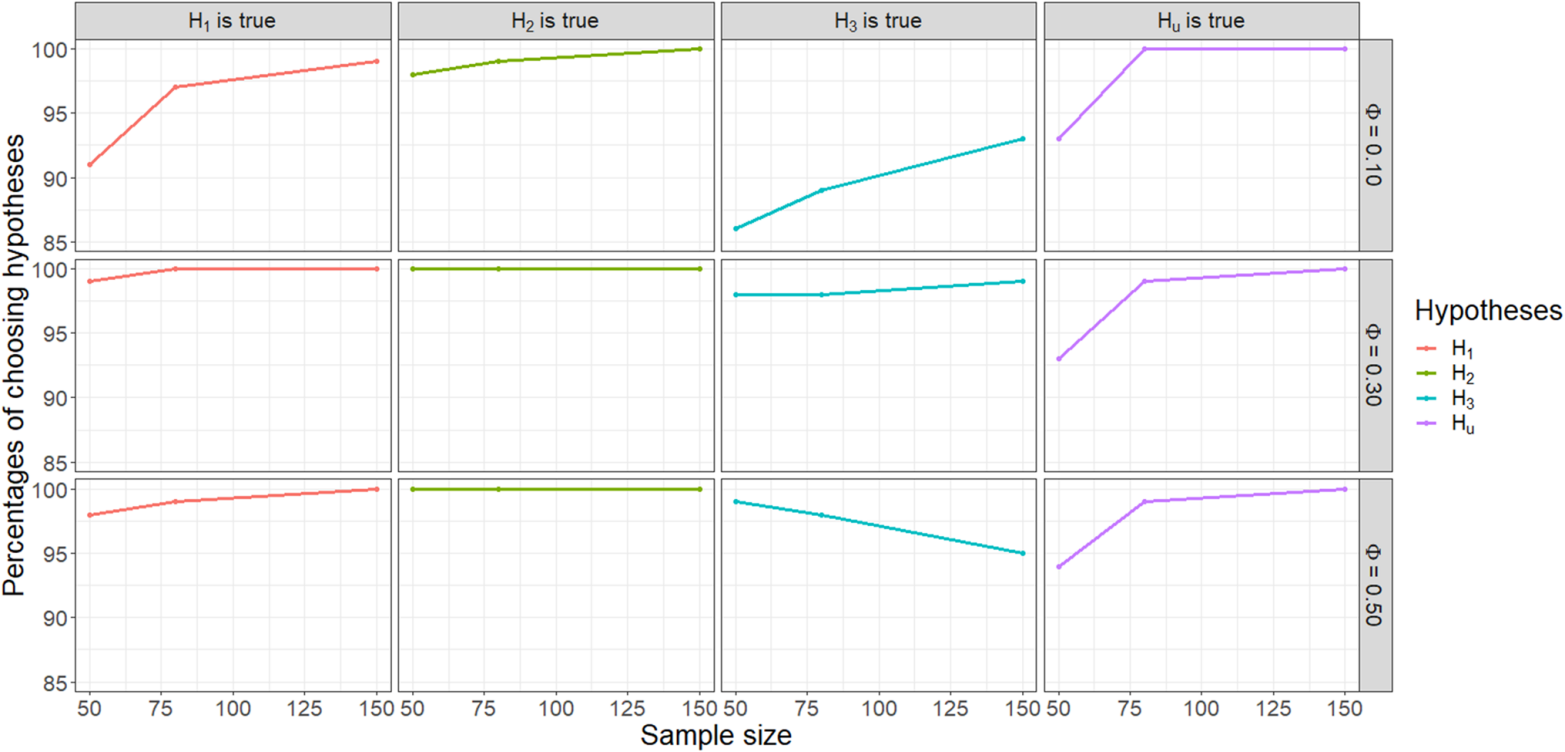
Percentage of times each hypothesis is chosen by GORICA under the selected true hypothesis with different values of sample size and effect size

In the simulation, we evaluate the four hypotheses containing non-linear restrictions on cell probabilities in (6) for four different cases. In each of the first three cases, one of the hypotheses $H_{1}$, $H_{2}$, and $H_{3}$ in (6) is chosen as the true hypothesis. In the last case, the population values of cell probabilities are only in accordance with the unconstrained hypothesis $H_{u}$. The sample size in the simulation is set to $N = 50, 80$, and 150. The values of cell probabilities in the contingency table are generated from a multinomial distribution using the expected value of Cramer’s V (Cohen, 1988) as the effect size measure. Since the number of rows in the contingency table is 2 and less than the number of columns, 4, the small, medium, and large values of Cramer’s V are defined as $\Phi = 0.10$, 0.30, and 0.50, respectively (Cohen, 1988, pp. 223–227). The population values of cell probabilities for the four cases are given in Table 7.

The simulation comprises the following steps:

1. Choose one of the hypotheses in (6) as the true hypothesis.
2. Set the effect size as $\Phi = 0.10, 0.30$, or 0.50.
3. Set the population values of cell probabilities based on the corresponding part in Table 7.
4. Set the sample size as $N = 50, 80$, or 150. Perform **Steps 4-9** until reaching $S = 1000$ successful simulation trials.
5. Generate the values of cell probabilities from the multinomial distribution based on the population values in **Step 3**.
6. Place the values from **Step 5** into a contingency table in $\texttt {table( )}$ format.
7. Set the zero(s) in the contingency table based on the place(s) of asterisk(s) (*) in Table 7.
8. Obtain the estimates of cell probabilities and their covariance matrix.
9. Apply our solution: Replace the zeros on the diagonal(s) of the covariance matrix with the value of the lowest variance in the same matrix.
10. Evaluate the hypotheses with the new covariance matrix using the $\texttt {gorica()}$ function in the $\texttt {gorica}$ package.
11. Calculate the percentages of times each of the hypotheses is chosen as the best hypothesis in the set with the smallest GORICA value (or the largest GORICA weight).

In each simulation condition, we expect a high percentage rate for the true hypothesis chosen in **Step 1**. We do not use a threshold for setting zeros in **Step 7**. The positions of asterisks (*) containing zeros are chosen in line with the restrictions of the hypotheses of interest. Note that the cell probability in the position of an asterisk in Table 7 is often the smallest cell probability in the contingency table for the corresponding simulation condition. If a problem is encountered during the simulation process in **Steps 4-9**, such as the covariance matrix in **Step 8** not being a positive definite matrix, we skip the corresponding iteration and move on to a new one. We continue the simulation until achieving $S = 1000$ successful simulation trials.

Figure 5 displays the percentage of times each hypothesis is chosen by GORICA under the selected true hypothesis with different values of sample size and effect size. It can be seen that GORICA performs quite satisfactorily in choosing the true hypothesis in the set. Even in the worst-case scenario where both the sample size and effect size are small (i.e., $N = 50$ and $\Phi = 0.10$), the true hypothesis $H_{1}$, $H_{2}$, $H_{3}$, or $H_{u}$ is chosen 91%, 98%, 86%, and 93% of the times, respectively. Considering all the other scenarios together, the true hypothesis is chosen at least 93% and at most 100% of the time. Note that increasing the sample size and/or effect size improves the performance of GORICA in choosing the best hypothesis in the set.

## GORICA illustrated

In this section, we apply GORICA to two examples. The first example is used to illustrate the evaluation of theory-based hypotheses containing non-linear restrictions on cell probabilities. The second example is used to exemplify the evaluation of theory-based hypotheses containing linear restrictions on cell probabilities in case of two high-dimensional contingency tables with empty cells due to sampling zeros.

### The gender by earned degrees example (continued)

Consider again the gender by earned degrees example from the introduction for which the true cell probabilities $(D = 8)$ and the data set $(N = 2403)$ were introduced earlier in Table 1.

#### Hypothesis specification

The hypotheses of interest for the gender by earned degrees example are formulated in (6).

**Table 8:** The estimates $\varvec{\hat{\eta }}$ and the covariance matrix $\varvec{\hat{\Sigma }_{\hat{\eta }}}$ of the estimates of the conditional cell probabilities for the gender by earned degrees example

		$$\varvec{\hat{\Sigma }_{\hat{\eta }}}$$			
$\varvec{\eta }$	$\varvec{\hat{\eta }}$	$\eta _{1}$	$\eta _{2}$	$\eta _{3}$	$\eta _{4}$
$\eta _{1}$	0.415	1.4e–4			
$\eta _{2}$	0.393	5.1e–6	3.7e–4		
$\eta _{3}$	0.463	5.2e–6	3.9e–6	2.8e–3	
$\eta _{4}$	0.500	5.8e–6	6.5e–5	–8.4e–6	5.2e–3

*Note.* $$\varvec{\eta } = (\eta _{1}, \eta _{2}, \eta _{3}, \eta _{4})^{\top } = (\frac{\pi _{21}}{\pi _{+1}},\frac{\pi _{22}}{\pi _{+2}}, \frac{\pi _{23}}{\pi _{+3}},\frac{\pi _{24}}{\pi _{+4}})^{\top }$$

#### Hypothesis evaluation

The MLEs of the conditional cell probabilities in (6) and their covariance matrix are given in Table 8. The order-restricted maximum log-likelihoods and penalties, the GORICA values, and the GORICA weights for the four hypotheses are given in Table 9.

#### Interpretation of the results

Based on the GORICA weights, hypotheses $H_{1}$, $H_{2}$, and $H_{3}$ are not weak hypotheses as they are $0.238/0.165\approx 1.44 > 1$, 0.415$/$0.165 $\approx$ 2.52 > 1, and $0.182 /0.165 \approx 1.10> 1$ times more supported than the unconstrained hypothesis $H_{u}$, respectively. Since at least one of the theory-based hypotheses is not a weak hypothesis, comparing their relative strengths is meaningful. Hypothesis $H_{2}$ is $0.415 /0.238 \approx 1.74 > 1$ and $0.415 /0.182 \approx 2.28 > 1$ times more supported than hypotheses $H_{1}$ and $H_{3}$, respectively. Therefore, it is concluded that, although all the hypotheses under consideration are supported by the data, hypothesis $H_{2}$ better fits the data when compared to the other two inequality-constrained hypotheses.

This subsection has shown that GORICA can easily be applied to evaluate hypotheses containing non-linear restrictions on cell probabilities. For detailed information on how to use the gorica package to evaluate the hypotheses in R, see the subsection “Example 1: Evaluating non-linear restrictions” in the online supplementary material. The next subsection illustrates the applicability of GORICA for high-dimensional contingency tables containing empty cells due to sampling zeros.

**Table 9:** The order-restricted maximum log-likelihoods $L(\varvec{\tilde{\eta }}_{m}|\varvec{\hat{\eta }}, \varvec{\hat{\Sigma }_{\hat{\eta }}})$, the penalties $PT_{m}(\varvec{\eta })$, the GORICA values $GORICA_{m}$, and the GORICA weights $w_{m}$ for hypothesis $H_{m}$ with $m = 1, 2, 3$, and u for the gender by earned degrees example

$H_{m}$	$L(\varvec{\tilde{\eta }}_{m}|\varvec{\hat{\eta }}, \varvec{\hat{\Sigma }}_{\varvec{\hat{\eta }}})$	$PT_{m}(\varvec{\eta })$	$GORICA_{m}$	$w_{m}$
$H_{1}$	9.214	2.584	–13.261	0.238
$H_{2}$	9.694	2.505	–14.377	0.415
$H_{3}$	8.544	2.182	–12.724	0.182
$H_{u}$	10.267	4.000	–12.535	0.165

### The eye-tracking example

**Fig. 6:**
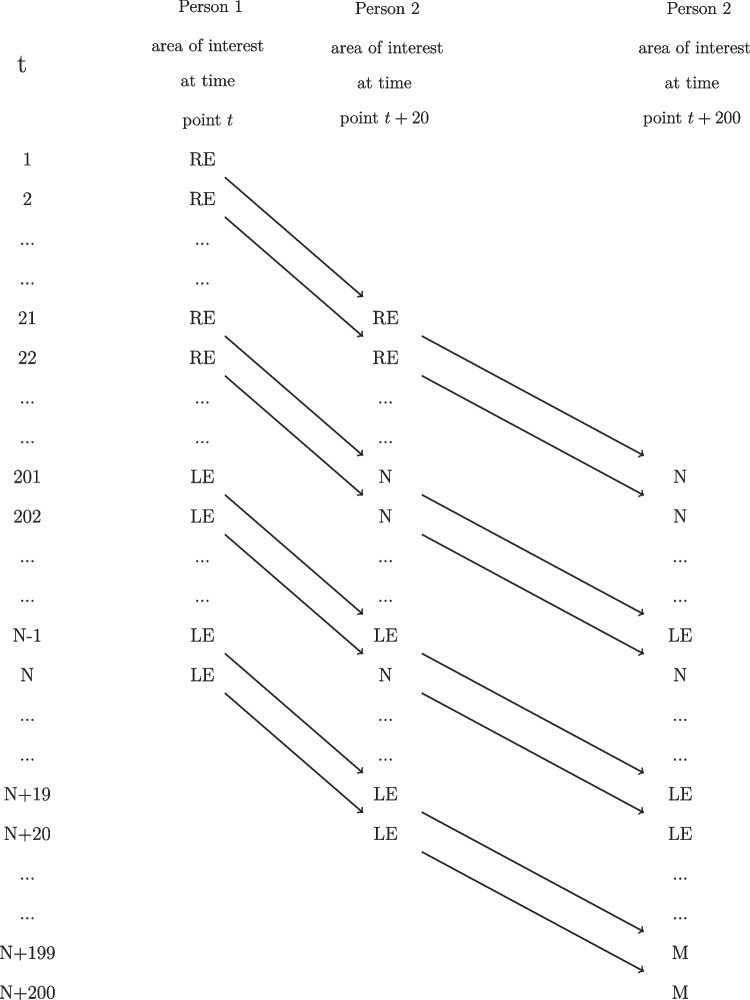
The eye-tracking dataset in the case of the gaze location of Person 1 influences the gaze location of Person 2

In this example, we illustrate the use of GORICA for restrictions on marginal cell probabilities in high-dimensional contingency tables containing empty cells. Examining marginal cell probabilities, that is, the cell probabilities marginalized (summed) over one or more variable(s) in a contingency table results in linear restrictions. Consider a study carried out to determine the relation between the gaze locations of two participants (i.e., Person 1 and Person 2) interacting with each other. The gaze location of Person 1 and Person 2 were tracked and recorded using the setup described in Hessels et al. (2017). This resulted in a series of 9051 time frames each representing 33.3 ms (i.e., a total of 301.7 s). The gaze location data are assigned to the categorical variable Area of Interest, defined as $\textrm{AOI} \in \{ 1 = \textrm{Nose}, 2 = \textrm{Mouth},$ $3 = \mathrm {Right \text Eye},$ $4 = \mathrm {Left\text Eye},$ $5 = \textrm{None}\}$. The levels denote the five areas of interest presented in Hessels et al. (2017) where the last level, None, is used to indicate that a gaze coordinate was collected but the gaze location was not on the other areas of interest. Table 10 shows the true cell probabilities ($D = 125$) and the corresponding data between brackets. The cell probability $\pi _{ijv}$ is the true probability of Person 1 looking at the *i*th level of AOI at time point *t*, Person 2 looking at the *j*th level of AOI at time point $t+20$, and Person 2 looking at the *v*th level of AOI at time point $t+200$ for $i, j, v = 1, 2, ..., 5$ and $t = 1, 2, ..., N = 8851$. As can be seen in Fig. 6, the data set contains $N+200$ time frames to investigate the gaze locations of the two persons at different time points.

**Table 10:** Population probabilities $\pi _{ijv}$ and observed cell frequencies between brackets for the eye-tracking example in the case of the gaze location of Person 1 influences the gaze location of Person 2

$\pi _{ijv}$		Person 2 at time point $t+200$				
Person 1 at time point *t*	Person 2 at time point $t+20$	Nose	Mouth	Right eye	Left eye	None
1 = Nose	Nose	$\pi _{111}$ (51)	$\pi _{112}$ (2)	$\pi _{113}$ (42)	$\pi _{114}$ (46)	$\pi _{115}$ (7)
	Mouth	$\pi _{121}$ (4)	$\pi _{122}$ (0)	$\pi _{123}$ (5)	$\pi _{124}$ (3)	$\pi _{125}$ (0)
	Right eye	$\pi _{131}$ (44)	$\pi _{132}$ (4)	$\pi _{133}$ (174)	$\pi _{134}$ (39)	$\pi _{135}$ (2)
	Left eye	$\pi _{141}$ (14)	$\pi _{142}$ (3)	$\pi _{143}$ (34)	$\pi _{144}$ (36)	$\pi _{145}$ (4)
	None	$\pi _{151}$ (4)	$\pi _{152}$ (1)	$\pi _{153}$ (26)	$\pi _{154}$ (11)	$\pi _{155}$ (0)
2 = Mouth	Nose	$\pi _{211}$ (1)	$\pi _{212}$ (0)	$\pi _{213}$ (1)	$\pi _{214}$ (2)	$\pi _{215}$ (0)
	Mouth	$\pi _{221}$ (0)	$\pi _{222}$ (0)	$\pi _{223}$ (0)	$\pi _{224}$ (0)	$\pi _{225}$ (0)
	Right eye	$\pi _{231}$ (5)	$\pi _{232}$ (0)	$\pi _{233}$ (4)	$\pi _{234}$ (2)	$\pi _{235}$ (0)
	Left eye	$\pi _{241}$ (1)	$\pi _{242}$ (0)	$\pi _{243}$ (3)	$\pi _{244}$ (0)	$\pi _{245}$ (0)
	None	$\pi _{251}$ (0)	$\pi _{252}$ (0)	$\pi _{253}$ (0)	$\pi _{254}$ (0)	$\pi _{255}$ (0)
3 = Right eye	Nose	$\pi _{311}$ (1006)	$\pi _{312}$ (112)	$\pi _{313}$ (921)	$\pi _{314}$ (495)	$\pi _{315}$ (91)
	Mouth	$\pi _{321}$ (81)	$\pi _{322}$ (1)	$\pi _{323}$ (79)	$\pi _{324}$ (22)	$\pi _{325}$ (2)
	Right eye	$\pi _{331}$ (1084)	$\pi _{332}$ (109)	$\pi _{333}$ (1909)	$\pi _{334}$ (397)	$\pi _{335}$ (134)
	Left eye	$\pi _{341}$ (486)	$\pi _{342}$ (42)	$\pi _{343}$ (419)	$\pi _{344}$ (220)	$\pi _{345}$ (33)
	None	$\pi _{351}$ (63)	$\pi _{352}$ (3)	$\pi _{353}$ (151)	$\pi _{354}$ (26)	$\pi _{355}$ (1)
4 = Left eye	Nose	$\pi _{411}$ (34)	$\pi _{412}$ (3)	$\pi _{413}$ (7)	$\pi _{414}$ (21)	$\pi _{415}$ (1)
	Mouth	$\pi _{421}$ (8)	$\pi _{422}$ (0)	$\pi _{423}$ (2)	$\pi _{424}$ (1)	$\pi _{425}$ (0)
	Right eye	$\pi _{431}$ (23)	$\pi _{432}$ (0)	$\pi _{433}$ (20)	$\pi _{434}$ (13)	$\pi _{435}$ (3)
	Left eye	$\pi _{441}$ (6)	$\pi _{442}$ (11)	$\pi _{443}$ (5)	$\pi _{444}$ (0)	$\pi _{445}$ (0)
	None	$\pi _{451}$ (0)	$\pi _{452}$ (0)	$\pi _{453}$ (3)	$\pi _{454}$ (0)	$\pi _{455}$ (0)
5 = None	Nose	$\pi _{511}$ (28)	$\pi _{512}$ (0)	$\pi _{513}$ (18)	$\pi _{514}$ (4)	$\pi _{515}$ (7)
	Mouth	$\pi _{521}$ (0)	$\pi _{522}$ (0)	$\pi _{523}$ (2)	$\pi _{524}$ (0)	$\pi _{525}$ (0)
	Right eye	$\pi _{531}$ (22)	$\pi _{532}$ (2)	$\pi _{533}$ (98)	$\pi _{534}$ (14)	$\pi _{535}$ (1)
	Left eye	$\pi _{541}$ (5)	$\pi _{542}$ (0)	$\pi _{543}$ (25)	$\pi _{544}$ (0)	$\pi _{545}$ (0)
	None	$\pi _{551}$ (2)	$\pi _{552}$ (0)	$\pi _{553}$ (0)	$\pi _{554}$ (0)	$\pi _{555}$ (0)

#### Hypothesis specification

The evaluation of interaction versus no interaction hypotheses may be tremendously useful in the study of gaze behavior in face-to-face interactions (see e.g., Brône and Oben, 2018; Hessels, 2020). In the study, the hypotheses of interaction state a specific interaction, where the gaze location of one participant guides the gaze location of the other participant, such that the second participant mimics where the first participant looks (i.e., the gaze location). This influence is more explicit at time frames that are relatively close to each other. In contrast, the hypotheses of no interaction state that the participants do not influence the gaze location of each other for a given time point. There are two hypotheses of interaction ($H_{1}$ and $H_{2}$) and two hypotheses of no interaction ($H_{3}$ and $H_{4}$). Hypothesis $H_{1}$ states that the probability of Person 1 at time point *t* and Person 2 at time point $t+20$ looking at the same area of interest with all possible levels at time point $t+200$ (i.e., $\pi _{11+}$ for nose, $\pi _{22+}$ for mouth, $\pi _{33+}$ for right eye, $\pi _{44+}$ for left eye, respectively) is larger than the probability of Person 1 at time point *t* and Person 2 at time point $t+200$ looking at the same area of interest with all possible levels at time point $t+20$ (i.e., $\pi _{1+1}$ for nose, $\pi _{2+2}$ for mouth, $\pi _{3+3}$ for right eye, $\pi _{4+4}$ for left eye, respectively):

7 $$\begin{aligned} H_{1}: \pi _{11+}>\pi _{1+1}, \pi _{22+}> \pi _{2+2}, \pi _{33+}> \pi _{3+3}, \pi _{44+} > \pi _{4+4}. \end{aligned}$$

The marginal cell probabilities in (7) represent the cell probabilities that are summed over the levels of one variable, for example, $\pi _{11+} = \pi _{111}+ \pi _{112}+ \pi _{113}+ \pi _{114}+ \pi _{115}$, that is, the probability of Person 1 at time point *t* looking at the nose and Person 2 at time point $t+20$ looking at the nose with all possible levels at time point $t+200$. Next, we provide the set of hypotheses: the four hypotheses of interest and the unconstrained hypothesis $H_{u}$. Subsequently, we describe hypotheses $H_{2}$, $H_{3}$, $H_{4}$, and the unconstrained hypothesis $H_{u}$ briefly.

8 $$\begin{aligned} &  H_{1}: \pi _{11+}>\pi _{1+1}, \pi _{22+}> \pi _{2+2}, \pi _{33+}> \pi _{3+3}, \pi _{44+}> \pi _{4+4}, \nonumber \\ &  H_{2}: \pi _{11+}>\pi _{1+1}, \pi _{22+}> \pi _{2+2}, \pi _{34+}> \pi _{3+4}, \pi _{43+} > \pi _{4+3}, \nonumber \\ &  H_{3}: \pi _{11+} = \pi _{1+1}, \pi _{22+} = \pi _{2+2}, \pi _{33+} = \pi _{3+3}, \pi _{44+} = \pi _{4+4}, \nonumber \\ &  H_{4}: \pi _{11+} = \pi _{1+1}, \pi _{22+} = \pi _{2+2}, \pi _{34+} = \pi _{3+4}, \pi _{43+} = \pi _{4+3}, \nonumber \\ &  H_{u}: \pi _{11+}, \pi _{1+1}, ..., \pi _{4+4}. \end{aligned}$$

Note that we reduce the number of the parameters from 124 to 12 (without loss of information) by estimating the 12 marginal cell probabilities, that is, $\varvec{\eta } = (\eta _{1}, \eta _{2}, ..., \eta _{12})^{\top }$ = $(\pi _{11+},\pi _{1+1}, ..., \pi _{4+4})^{\top }.$

**Table 11:** The estimates $\varvec{\hat{\eta }}$ and the adjusted covariance matrix $\varvec{\hat{\Sigma }}^{adj}_{\varvec{\hat{\eta }}}$ for the eye-tracking example

In the case of the gaze location of Person 1 influences the gaze location Person 2													
		$$\varvec{\hat{\Sigma }}_{\varvec{\hat{\eta }}}$$											
$\varvec{\eta }$	$\varvec{\hat{\eta }}$	$\eta _{1}$	$\eta _{2}$	$\eta _{3}$	$\eta _{4}$	$\eta _{5}$	$\eta _{6}$	$\eta _{7}$	$\eta _{8}$	$\eta _{9}$	$\eta _{10}$	$\eta _{11}$	$\eta _{12}$
$\eta _{1}$	0.017	1.8e–6											
$\eta _{2}$	0.013	6.2e–7	1.5e–6										
$\eta _{3}$	0.000	0.000	0.000	3.2e–7									
$\eta _{4}$	0.000	0.000	0.000	0.000	3.2e-7								
$\eta _{5}$	0.410	–1.1e–6	–2.9e–7	0.000	0.000	2.8e–5							
$\eta _{6}$	0.393	–5.8e–7	–4.9e–7	0.000	0.000	6.7e–6	2.6e–5						
$\eta _{7}$	0.136	–2.6e–7	–4.2e–	0.000	0.000	–6.4e–6	–3.1e–7	1.3e–5					
$\eta _{8}$	0.131	–2.3e–7	–2.5e–7	0.000	0.000	–1.5e–6	–5.3e–6	4.0e–7	1.2e–5				
$\eta _{9}$	0.007	–1.4e–8	–4.6e–8	0.000	0.000	–2.2e–7	–2.1e–7	2.8e–8	–2.0e–7	7.2e–7			
$\eta _{10}$	0.004	–5.6e–8	–5.5e–9	0.000	0.000	–2.0e–8	–1.8e–7	–3.1e–8	–6.2e–8	2.4e–7	4.5e–7		
$\eta _{11}$	0.002	1.6e-8	–1.0e–8	0.000	0.000	–1.2e–8	–1.1e–7	3.9e–8	–9.0e–8	8.9e–9	5.4e–8	3.2e–7	
$\eta _{12}$	0.004	–1.8e–8	–2.2e–8	0.000	0.000	–2.4e–7	–2.3e–7	–1.5e–8	–1.2e–7	1.6e–7	–1.2e–9	–7.4e–9	4.2e–7

*Note.* $$\varvec{\eta }= (\eta _{1}, \eta _{2}, \eta _{3}, \eta _{4},\eta _{5},\eta _{6},\eta _{7},\eta _{8},\eta _{9},\eta _{10},\eta _{11},\eta _{12})^{\top } = (\pi _{11+},\pi _{1+1},\pi _{22+},\pi _{2+2},\pi _{33+},\pi _{3+3},\pi _{34+},\pi _{3+4},$$ $$\pi _{43+},\pi _{4+3},$$ $$\pi _{44+},\pi _{4+4})^{\top }$$

Hypothesis $H_{2}$ states that the expectation in $H_{1}$ remains valid for only the areas of interest nose and mouth, and because Person 1 and Person 2 sit across from each other the left eye and the right eye should be shifted. That is, the probability of Person 1 at time point *t* looking at the left eye and Person 2 at time point $t+20$ looking at the right eye with all possible levels at time point $t+200$ (i.e., $\pi _{34+}$) is larger than the probability of Person 1 at time point *t* looking at the left eye and Person 2 at time point $t+200$ looking at the right eye with all possible levels at time point $t+20$ (i.e., $\pi _{3+4}$) and vice versa (i.e., $\pi _{43+}$ compared to $\pi _{4+3}$). Compared to hypothesis $H_{1}$, the no-interaction hypothesis $H_{3}$ specifies that the gaze location of Person 1 does not influence the gaze location of Person 2, and therefore, the corresponding marginal cell probabilities in hypothesis $H_{1}$ are set equal to each other. Similarly, the no-interaction hypothesis $H_{4}$ specifies that the gaze location of Person 1 does not influence the gaze location of Person 2, and therefore, the marginal cell probabilities in hypothesis $H_{2}$ are set equal to each other. The unconstrained hypothesis $H_{u}$ does not restrict any parameters, and thus, covers the relationships between marginal cell probabilities that are represented by hypotheses $H_{1}$, $H_{2}$, $H_{3}$, and $H_{4}$, and all the other possibilities. This hypothesis is used to avoid choosing a weak hypothesis out of the four hypotheses.

During the experiment, both individuals may influence each other’s gaze location. Therefore, a data set ($N=8851$ video frames) is analyzed under two possible scenarios. For both scenarios, the same hypotheses, namely those in (8), are evaluated. In the first scenario, as mentioned, the gaze location of Person 1 is considered to influence the gaze location of Person 2, such that Person 2 follows Person 1. In the second scenario, the gaze location of Person 2 is considered to influence the gaze location of Person 1, and therefore, the roles of Person 1 and Person 2 are turned around, see the section “The eye-tracking example for the second scenario” in the Online Supplementary Material.

#### Hypothesis evaluation

In Table 10, the true cell probabilities and observed cell frequencies are displayed. Notably, the table contains many empty cells. The corresponding MLEs and their covariance matrix in terms of the reparameterized cell probabilities, that is, the marginal cell probabilities in (8), are given in Table 11. In this scenario, $\hat{\eta }_{3} = \hat{\pi }_{22+} = 0$ and $\hat{\eta }_{4} = \hat{\pi }_{2+2} = 0$ because of the sampling zeros. Therefore, the zeros in the third and fourth diagonal entries of the covariance matrix are replaced by the smallest diagonal entry in the same matrix. In Table 12, the order-restricted maximum log-likelihoods and penalties, the GORICA values, and the GORICA weights for the five hypotheses are displayed.

#### Interpretation of the results

Based on the GORICA weights in Table 12, the no-interaction hypotheses $H_{3}$ and $H_{4}$ do not receive any support from the data set (i.e., hypotheses $H_{3}$ and $H_{4}$ have nearly zero GORICA weights, which are significantly less than that of the unconstrained hypothesis $H_{u}$). The interaction hypothesis $H_{1}$ receives $0.182 /0.101 \approx 1.80$ times more support from the data than the unconstrained hypothesis $H_{u}$. The interaction hypothesis $H_{2}$ receives a considerable amount of support from the data: Hypothesis $H_{2}$ is $0.712 /0.101 \approx 7.05$ times more supported than the unconstrained hypothesis $H_{u}$. Hence, hypothesis $H_{2}$ is a strong hypothesis. Notably, the restrictions of hypothesis $H_{2}$ are in accordance with the data since the order-restricted maximum log-likelihood value for $H_{2}$ is equal to that for the unconstrained hypothesis $H_{u}$. Thus, the order-restricted MLEs for $H_{2}$ are equal to the MLEs for the unconstrained hypothesis $H_{u}$. Additionally, hypothesis $H_{2}$ is $0.712 /0.182 \approx 3.91$ times more supported than hypothesis $H_{1}$ and it is supported many more times than hypotheses $H_{3}$ and $H_{4}$. Consequently, hypothesis $H_{2}$ is selected as the best hypothesis among the four theory-based hypotheses. In general, it seems that the interaction hypotheses are more supported by the data than the no-interaction hypotheses.

In conclusion, using the log-linear model in evaluating hypotheses for a $5 \times 5 \times 5$ contingency table involves 124 parameters in total, including two-way and three-way interaction terms, which are difficult to interpret by researchers. The use of theory-based hypotheses facilitates the interpretation. By using GORICA, only 12 $\eta$ parameters and their covariance matrix need to be estimated to evaluate the hypotheses in (8). This then renders a quantification of the support for the hypotheses of interest. It has been shown via our simulation that GORICA is not affected by the negative effects of empty cells due to sampling zeros on parameter estimation when evaluating theory-based hypotheses for contingency tables. For detailed information on how to use the $\texttt {gorica}$ package to evaluate hypotheses in the presence of sampling zeros, see the subsection “Example 2: Evaluating hypotheses in the presence of sampling zeros” in the Online Supplementary Material.

**Table 12:** The order-restricted maximum log-likelihoods $L(\varvec{\tilde{\eta }}_{m}|\varvec{\hat{\eta }},$ $\varvec{\hat{\Sigma }}^{adj}_{\varvec{\hat{\eta }}})$, the penalties $PT_{m}(\varvec{\eta })$, the GORICA values $GORICA_{m}$, and the GORICA weights $w_{m}$ for hypothesis $H_{m}$ with $m = 1, 2, 3, 4$, and *u* in the case of the gaze location of Person 1 influences the gaze location Person 2

$H_{m}$	$L(\varvec{\tilde{\eta }}_{m}|\varvec{\hat{\eta }}, \varvec{\hat{\Sigma }}^{adj}_{\varvec{\hat{\eta }}})$	$PT_{m}(\varvec{\eta })$	$GORICA_{m}$	$w_{m}$
$H_{1}$	67.228	9.983	–114.490	0.182
$H_{2}$	68.654	10.044	–117.220	0.712
$H_{3}$	59.779	8.000	–103.559	0.001
$H_{4}$	61.481	8.000	–106.96	0.004
$H_{u}$	68.654	12.000	–113.308	0.101

## Discussion

Testing hypotheses of no relationship is a common analysis in log-linear models. Such hypotheses containing only equality restrictions can be evaluated using AIC (Akaike, 1973, 1974). However, AIC cannot be used to assess researchers’ expectations, if they contain inequality restrictions. GORICA (Altinisik et al., 2021) fills this gap in the presence of inequality restrictions and reduces to AIC if the hypothesis of interest contains only equality restrictions. This paper introduces GORICA in the context of contingency tables.

GORICA is an approximation method that assumes a large sample. Therefore, GORICA should be used with caution when using it with small samples in the context of contingency tables. Additionally, GORICA necessitates the estimates of (the functions of) cell probabilities and their covariance matrix obtained by nonparametric bootstrapping (Efron and Tibshirani, 1993, p. 45). It is a well-known fact that nonparametric bootstrapping assumes that the observed sample is a good representative of the population of interest, often requiring a sufficient sample size (Hox et al., 2010). Our method works in such a way that it will not be affected by empty cells. The method is implemented in the gorica package, see the section “Evaluating hypotheses using the gorica package” in the Online Supplementary Material.

Two examples were analyzed to illustrate the applicability of GORICA. This new method enables researchers to evaluate theory-based hypotheses for (sparse) high-dimensional contingency tables. The relevant R code are available on the Web at **GitHub**.

## Supplementary Information

Below is the link to the electronic supplementary material.

Supplementary file 1 (pdf 536 KB)

## Data Availability

The relevant data and R code are available on https://github.com/rebeccakuiper/GORICA-for-contingency-tables.

## Appendix A

### Obtaining $\hat{\Sigma }_{\hat{\theta }}$ with nonparametric bootstrapping

GORICA (Altinisik et al., 2021) uses the large sample theory (Fisher, 1922) to approximate the log-likelihood of the data using a normal distribution:

9 $$\begin{aligned} L (\varvec{\tilde{\theta }}_{m} | \varvec{\hat{\theta }} ,\varvec{\hat{\Sigma }}_{\varvec{\hat{\theta }}} )= -\frac{K}{2} \textrm{log} (2\pi ) - \frac{1}{2} \textrm{log} |\varvec{\hat{\Sigma }}_{\varvec{\hat{\theta }}} |- \mathrm {\frac{1}{2}}(\varvec{\hat{\theta }}-\varvec{\tilde{\theta }}_{m})^{T}(\varvec{\hat{\Sigma }}_{\varvec{\hat{\theta }}})^{\mathrm {-1}}(\varvec{\hat{\theta }} - \varvec{\tilde{\theta }}_{m}). \end{aligned}$$

In contingency tables, the parameters can be either cell probabilities^4^, that is, $\varvec{\theta } = \varvec{\eta } = \varvec{\pi }^{-1}$, or the functions of cell probabilities (stated otherwise, reparameterized cell probabilities), that is, $\varvec{\theta } = \varvec{\eta } = g(\varvec{\pi })$. GORICA uses the MLEs of (the functions of) cell probabilities, that is, $\hat{\theta }$s. These values need to be obtained from the observed contingency tables. For example, consider the hypothesis $H_{1}: \frac{\pi _{21}}{\pi _{+1}} > \{\frac{\pi _{22}}{\pi _{+2}},\frac{\pi _{23}}{\pi _{+3}},\frac{\pi _{24}}{\pi _{+4}}\}$ in (1) for which $\varvec{\theta } = (\theta _{1}, \theta _{2}, \theta _{3}, \theta _{4})^\top = (\frac{\pi _{21}}{\pi _{+1}}, \frac{\pi _{22}}{\pi _{+2}}, \frac{\pi _{23}}{\pi _{+3}}, \frac{\pi _{24}}{\pi _{+4}})^\top$, and let us denote $D = I \times J = 2 \times 4 = 8$ as the number of $\pi$s and $K = 4$ as the number of $\eta$s. The MLEs of the $\theta$s are $\varvec{\theta } = (\hat{\theta }_{1}, \hat{\theta }_{2}, \hat{\theta }_{3}, \hat{\theta }_{4})^\top = (\frac{\hat{\pi }_{21}}{\hat{\pi }_{+1}}, \frac{\hat{\pi }_{22}}{\hat{\pi }_{+2}}, \frac{\hat{\pi }_{23}}{\hat{\pi }_{+3}}, \frac{\hat{\pi }_{24}}{\hat{\pi }_{+4}})^\top$. Notably, we used the invariance property of the MLEs, that is, if $\varvec{\hat{\pi }}$ contains the MLEs for $\varvec{\pi }$, then $\varvec{\hat{\theta }} = g(\varvec{\hat{\pi }})$ contains the MLEs for $\varvec{\theta } = g(\varvec{\pi })$. Although the MLEs of $\theta$s can easily be obtained using their invariance property in the context of contingency tables, obtaining the covariance matrix of these estimates, the $\varvec{\hat{\Sigma }}_{\varvec{\hat{\theta }}}$, is problematic. The standard software (e.g., R, SAS, Python, Mplus) uses maximum likelihood estimation as the default estimation method and does not render the covariance matrix of the MLEs of (the reparameterized) cell probabilities in the context of contingency tables. Therefore, nonparametric bootstrapping (Efron and Tibshirani, 1993, p. 45) is used to obtain the covariance matrix $\varvec{\hat{\Sigma }}_{\varvec{\hat{\theta }}}$. We provide the general estimation procedure below.

For simplicity of notation, the observed cell frequencies in a multidimensional contingency table are stacked together in a $D \times 1$ vector. For example, in case of the three-way contingency table introduced in Table 3, the vector of the observed cell frequencies is defined as $\varvec{y} = (y_{111}, ..., y_{ijv}, ..., y_{IJV})^\top = (y_{1}, ..., y_{d}, ..., y_{D})^\top$, where $N = \sum _{d=1}^{D}y_{d}$ is the sum of the cell frequencies. Then, nonparametric bootstrapping to obtain $\varvec{\hat{\Sigma }}_{\varvec{\hat{\theta }}}$ consists of the following steps:

1. Create $y_{d}$ data points with the value *d* for $d = 1, 2, ..., D$. Note that the data contain numbers from 1 to *D*.
2. Draw *B* independent bootstrap samples^5^ of size *N* with replacement from the data such that each bootstrap sample represents one contingency table for which the entries are $y_{bd}$, that is, the cell frequency $d = 1, 2, ..., D$ in bootstrap sample $b = 1, 2, ..., B$.
3. Each of the *B* bootstrap sample leads to the MLEs of the *K* elements in $\varvec{\eta }$, denoted by $\varvec{\hat{\theta }}_{bk} = g(\hat{\pi }_{b1},..., \hat{\pi }_{bD})^\top$, where $\hat{\pi }_{bd} = \frac{y_{bd}}{N}$ is the *d*th cell probability in the *b*th bootstrap sample for $b = 1, 2, ..., B$. To illustrate, consider the example above where $D = 2 \times 4 = 8$, $K = 4$, and $\varvec{\theta } = (\theta _{1}, \theta _{2}, \theta _{3}, \theta _{4})^\top = (\frac{\pi _{21}}{\pi _{+1}}, \frac{\pi _{22}}{\pi _{+2}}, \frac{\pi _{23}}{\pi _{+3}}, \frac{\pi _{24}}{\pi _{+4}})^\top$. The MLE of parameter $\theta _{1}$ in the *b*th bootstrap sample is $\hat{\theta }_{b1} = \frac{\hat{\pi }_{b21}}{\hat{\pi }_{b+1}}$.
4. Obtain the covariance matrix of $\hat{\theta }$s where element $(k\text {, }k^{\prime })$ is calculated as:
  10 $$\begin{aligned} \varvec{\hat{\Sigma }}_{\varvec{\hat{\theta }}, k k^{\prime } }\! =\! \frac{1}{B-1}\sum _{b=1}^{B}(\hat{\theta }_{bk}-\overline{\theta }_{k})(\hat{\theta }_{bk^{\prime }}-\overline{\theta }_{k^{\prime }}), \text { for } k\!=\!1, 2, ..., K, \end{aligned}$$
  with $\overline{\theta }_{k}$ the mean of the *B* bootstrap values for the *k*th parameter.

Once the MLEs of the $\theta$ parameters ($\varvec{\hat{\theta }}$) are obtained using the original contingency table, and their covariance matrix ($\varvec{\hat{\Sigma }}_{\varvec{\hat{\theta }}}$) is obtained using the nonparametric estimation procedure above, the values of $\varvec{\tilde{\theta }}$ for $m = 1, 2, ..., M$ are estimated by

11 $$\begin{aligned} {\varvec{\tilde{\theta }}}_{m} = {\underset{\theta \in {H_{m}}}{\arg \min }} (\varvec{\hat{\theta }}-\varvec{\theta })^{\top }(\varvec{\hat{\Sigma }}_{\varvec{\hat{\theta }}})^{-1}(\varvec{\hat{\theta }}-\varvec{\theta }). \end{aligned}$$

The right-hand side of (11) represents the quadratic function which, needs to be minimized accounting for the (in)equality constraints in hypothesis $H_{m}$. In the gorica package, this is done using a quadratic programming algorithm, namely the solve.QP subroutine of the quadprog package (Turlach, 2014, pp. 2–4).

## Appendix B

### GORICA weights

After computing the GORICA values for each hypothesis separately, these values can be used to calculate the GORICA weights (Altinisik et al., 2021, p. 603). Comparable to the Akaike weights (Burnham and Anderson, 2002, p. 75), GORICA weight of hypothesis $H_{m}$ is defined as:

12 $$\begin{aligned} {w_{m}=\frac{\text {exp} \{{-\frac{1}{2}}\textrm{GORICA}_{m}\}}{{\sum _{m' = {1}}^{M}}\text {exp}\{{-\frac{1}{2}\text {GORICA}}_{m'}\}}}, \end{aligned}$$

where $w_{m}$ can take a value between 0 and 1 and the $w_{m}$s sum to one. The GORICA weight is utilized to quantify the support in the data for hypothesis $H_{m}$ compared to the set of *M* hypotheses.

Consider hypotheses $H_{1}, H_{2}$, and the unconstrained hypo-thesis $H_{u}$ with the GORICA weights 0.84, 0.04, and 0.12, respectively. Hypothesis $H_{1}$ is the best hypothesis since it has the highest GORICA weight in the set. Hypothesis $H_{1}$ is not a weak hypothesis since $H_{1}$ is $0.84/0.12 = 7$ times more supported by the data compared to $H_{u}$. In contrast, hypothesis $H_{2}$ is a weak hypothesis, that is, a hypothesis that is not supported by the data. Note that the unconstrained hypothesis $H_{u}$ is $0.12/0.04 = 3$ times more supported by the data compared to hypothesis $H_{2}$. Hypothesis $H_{1}$ better fits the data than hypothesis $H_{2}$, because it is $0.84/0.04 = 21$ times more supported by the data compared to $H_{2}$.

## Footnotes

1 Readers who are more in favor of the Bayesian framework instead of the information-theoretical one are referred to Klugkist et al. (2010), who evaluate theory-based hypotheses in the context of contingency tables using a model selection criterion called Bayes factor (Kass, 1993; Kass & Raftery, 1995)

2 When there is only one hypothesis of interest, in line with the suggestion made by Vanbrabant et al. (2020), we advise evaluating the hypothesis against its complement. This complement represents all the other theories $\textit{not}$ covered by the hypothesis, thus acting like a competing hypothesis. If multiple hypotheses exist in the set, we recommend including the unconstrained hypothesis $H_{u}$, which is the default option used as a failsafe in the gorica package.

3 The number of parameters that are not restricted by the hypotheses in the set has no effect on the penalty since this number is a fixed constant for each hypothesis under consideration (Altinisik et al., 2021, p. 620).
