# Supplementary material for: An AIC-type information criterion evaluating theory-based hypotheses for contingency tables
Source: Behav Res Methods. 2025 Jan 22;57(2):70. doi: 10.3758/s13428-024-02570-6 (PMC11754365; doi:10.3758/s13428-024-02570-6)
Supplement: Supplementary file 1 — (pdf 536 KB) [file 13428_2024_2570_MOESM1_ESM.pdf]

Supplementary Material

In this supplementary material, we first discuss the reasons for using the quadratic non-linear optimization without including the bound restrictions on ηs instead of the non-linear optimization with including the bound restrictions on πs. Second, we provide numerous examples for various hypotheses to contrast our solution for calculating the penalty without bound restrictions with the non-linear optimization with bound restrictions. We show that our solution does not influence the relative differences between penalties in the original scale under various circumstances. Third, we elaborate on the technical details on how to evaluate hypotheses in the presence of empty cells due to structural zeros. We propose a technique which involves rewriting the hypotheses of interest in case of empty cells due to structural zeros. Then, we provide a brief guidance and summary table on how to make decisions in rewriting hypotheses. Fourth, we revisit the Eye-Tracking example but this time for the second scenario in which the gaze location of Person 2 is considered to influence that of Person 1. Finally, we explain how to reproduce the results in the main text for the gender by earned degrees and Eye tracking examples using the `gorica` package in `R`.

Sampling with or without Bound Restrictions

The application of the non-linear optimization with bound restrictions is a cumbersome task in our context. As mentioned in the main text on page 17, the non-linear optimization with bound restrictions requires the specification of all restrictions on πs. For instance, the evaluation of the bounded unconstrained hypothesis H_u for a $5 \times 5 \times 5$ contingency table necessitates restrictions on all 125 cell probabilities taking into account that they sum to one. Additionally, it may not always be immediately obvious which bound restrictions hold after reparameterizing cell probabilities into ηs. For instance, the marginal cell probabilities (see Table 2 in the main text) and the local, marginal, and conditional odds ratios (see Table 3 in the main text) are always larger than zero for data without

empty cells.[1] Hypotheses containing these probabilities or ratios thus involve the bound restrictions $\boldsymbol{\eta} > 0$. In the same manner, since conditional cell probabilities (see Table 2 in the main text) are always between zero and one, hypotheses containing these probabilities involve the bound restrictions $0 < \boldsymbol{\eta} < 1$. Hence, it is imperative to exercise caution in determining the necessary bound restrictions while sampling with bound restrictions.

We have made the decision to employ the quadratic non-linear optimization without including the bound restrictions on ηs. The implementation of the quadratic non-linear optimization without bound restrictions on ηs is simpler and necessitates less computation time than the non-linear optimization with bound restrictions on πs. It also improves the usability of our method, because the user of the `gorica` package does not need to decide on and specify bound restrictions.

In the next section, we discuss the calculation of the penalty using the non-linear optimization with including the bound restrictions on $\boldsymbol{\pi}$ and the quadratic non-linear optimization without including the bound restrictions on $\boldsymbol{\eta}$. We compare the relative differences in penalties in both scenarios when evaluating different types of hypotheses containing linear or non-linear restrictions on cell probabilities. We demonstrate that our solution does not have an impact on the relative disparities among penalties in the original scale under diverse circumstances.

Comparing Penalty Weights between Non-linear Optimizations

We provide multiple examples (from hypotheses containing linear or non-linear restrictions on cell probabilities) to illustrate the comparison of relative differences in penalties. For this purpose, we use the gender by earned degrees data in Table 1 (in the

[1]Empty cells in contingency tables may cause some of ηs to be estimated as zero, one, or inestimable. Empty cells can occur in contingency tables due to sampling zeros or structural zeros. We elaborate on the evaluation of hypotheses in both scenarios, see the section “Problem B: Empty Cells due to Sampling Zeros” in the main text and the section “Hypotheses Evaluation in the Presence of Structural Zeros” in this supplementary material.

main text) and the hypotheses in Table 2 (in the main text) in the context of the 2×4 contingency table. We compare each of the hypotheses with the corresponding unconstrained hypothesis H_u using both the non-linear optimization method with including the bound restrictions on $\boldsymbol{\pi}$ and the quadratic non-linear optimization method without including the bound restrictions on $\boldsymbol{\eta}$ for $c = 1$ and $c = 100000$.

The relative differences in penalties should be invariant to the scaling of the covariance matrix determined by the value of c, as the penalty is a measure that is independent of the data. In **Tables 1** and **2**, the penalties (and penalty weights) are given for hypotheses with non-marginal and marginal cell probabilities, respectively. For $c = 1$, the penalty weights between the two non-linear optimization methods for hypotheses containing marginal and non-marginal cell probabilities are quite close to each other. It can be seen that if the hypotheses contain linear restrictions on cell probabilities, the penalty weight obtained by the non-linear optimization with including the bound restrictions on $\boldsymbol{\pi}$ varies with c. The task of optimizing c in a manner that best aligns with the feasible space is arduous in our context, and we lack any predetermined criteria for this purpose. In contrast, the penalty weight obtained by the quadratic non-linear optimization without including the bound restrictions on $\boldsymbol{\eta}$ is invariant with c. Sampling without bound restrictions is also simpler and requires less computation time. Therefore, we concluded that the quadratic non-linear optimization after reparameterizing cell probabilities provides reasonable penalties and it is more preferable than the non-linear optimization with including the bound restrictions on $\boldsymbol{\pi}$ when hypotheses contain linear restrictions on cell probabilities.

In **Tables 3** and **4**, the penalties (and penalty weights) are displayed for hypotheses with conditional cell probabilities and local odds ratios, respectively, as being representatives of hypotheses containing non-linear restrictions on cell probabilities. When hypotheses contain non-linear restrictions on cell probabilities, the penalty weight obtained by the non-linear optimization method with including the bound restrictions on $\boldsymbol{\pi}$ may or

may not change significantly with c. It appears that when hypotheses contain non-stringent non-linear restrictions on cell probabilities, the penalty weight obtained by this non-linear optimization method does not change much with c (e.g., hypothesis H_2 in **Table 3** and hypotheses H_1 and H_2 in **Table 4**). For the hypotheses containing stringent non-linear restrictions on cell probabilities, the penalty weight obtained by the non-linear optimization method varies widely with c (e.g., hypothesis H_1 in **Table 3**). Note that regardless of whether the hypothesis contains stringent or non-stringent non-linear restrictions on cell probabilities, the penalty weight obtained by the quadratic non-linear optimization method without including the bound restrictions on $\boldsymbol{\eta}$ does not vary with c. The quadratic non-linear optimization method produces close penalty weights when compared to the non-linear optimization method with including the bound restrictions on $\boldsymbol{\pi}$ for either $c = 1$ or $c = 100000$. Therefore, we concluded that the latter approach without bound restrictions is preferable to the former approach with bound restrictions, not only for hypotheses with linear restrictions on cell probabilities, but also for hypotheses with non-linear restrictions on cell probabilities.

In the next section, we propose a solution compatible with the quadratic non-linear optimization method that can be used to handle empty cells due to structural zeros in contingency tables.

Hypotheses Evaluation in the Presence of Structural Zeros

Structural zeros in a contingency table are always zero. Dealing with empty cells due to structural zeros requires a completely different solution than those caused by sampling zeros. When some of the ηs are estimated as zero or one, or cannot be estimated due to structural zeros, the solution is to rewrite the hypothesis of interest. In the following two subsections, we elaborate on the solution in cases where $\hat{\eta} = 0$, $\hat{\eta} = 1$, or η is inestimable.

Rewriting hypotheses based on $\hat{\eta} = 0$

When some of the ηs are estimated as zero because of empty cell(s) due to structural zeros (but not sampling zeros), the solution is to rewrite the hypothesis of interest. This requires defining $\tilde{\boldsymbol{\eta}}_m = \{\tilde{\boldsymbol{\eta}}_E, \tilde{\boldsymbol{\eta}}_F\}$, where $\tilde{\boldsymbol{\eta}}_E$ and $\tilde{\boldsymbol{\eta}}_F$ represent the order-restricted MLEs of $\boldsymbol{\eta_E}$ and $\boldsymbol{\eta_F}$, which correspond to the ηs that are estimated as zero because of the Empty cells and as non-zero for the Full (or non-empty) cells, respectively.

The rewritten hypothesis needs adjusted restriction matrices $\boldsymbol{S_m^{adj}}$ and/or $\boldsymbol{R_m^{adj}}$ and may require adjusted constants $\boldsymbol{s_m^{adj}}$ and/or $\boldsymbol{r_m^{adj}}$, as will be described in the 2-step procedure below which is embedded in the `gorica` package. To keep the exposition simple, we provide this procedure in terms of hypotheses containing only inequality restrictions, which can be extended for hypotheses containing equality restrictions in the same manner. Subsequently, we will give three examples for the three different scenarios discussed in the procedure.

1. Discard the rows and columns from $\hat{\boldsymbol{\Sigma}}_{\hat{\boldsymbol{\eta}}}$ for which all elements are zero. Then, discard the corresponding ηs and the corresponding columns from the restriction matrix $\boldsymbol{R}_m$ (see Equation 4 in the main text), which leads to $\boldsymbol{\eta}^{adj}$ and $\boldsymbol{R}_m^{adj}$, respectively. Notably, in this way, $\boldsymbol{\eta}_E$ (i.e., the vector of ηs that are estimated as zero) is discarded from the restrictions. Next, we have to focus on $\boldsymbol{R}_m^{adj}$ to investigate whether discarding $\boldsymbol{\eta}_E$ from the restrictions requires an adjustment for the constants in the hypotheses.

2. Check the rows of $\boldsymbol{R}_m^{adj}$ to determine whether the constants in $\boldsymbol{r}_m$ have to be adjusted as well.

   (a) If there are no row(s) in $\boldsymbol{R}_m^{adj}$ with only zeros, $\boldsymbol{r}_m$ does not need adjustment and $\tilde{\boldsymbol{\eta}}_E = \hat{\boldsymbol{\eta}}_E = 0$.

   (b) If there are row(s) in $\boldsymbol{R}_m^{adj}$ with only zeros and the corresponding constants are

      i. non-positive, $\boldsymbol{r}_m$ does not need adjustment, because $\tilde{\boldsymbol{\eta}}_E = \hat{\boldsymbol{\eta}}_E = 0$ is in agreement with the restrictions in H_m.

ii. positive, $\boldsymbol{r}_m$ has to be adjusted, since $\tilde{\boldsymbol{\eta}}_E = \hat{\boldsymbol{\eta}}_E = 0$ is not in agreement with the restrictions in H_m. Therefore, first calculate the $\tilde{\boldsymbol{\eta}}_E$ that is in accordance with the restrictions in H_m, and then adjust $\boldsymbol{r}_m$ accordingly. This is done as follows:

- Calculate the values $\boldsymbol{q} = \{\boldsymbol{q}_E, \boldsymbol{q}_F\}$ for which $\boldsymbol{R}_m\boldsymbol{q} = \boldsymbol{r}_m$.

The $\boldsymbol{q}_E$ and $\boldsymbol{q}_F$ represent values on the boundary of the parameter space defined by the restrictions in H_m. These q values are obtained using a least distance programming algorithm, the `ldei` subroutine of the `limSolve` package (**Soetaert et al., 2014,** pp. 11-13) in `R`. The subroutine `ldei` finds the least distance in the sense that the sum of squared qs is minimal:

$$
\begin{aligned}
&\min(\boldsymbol{q} \circ \boldsymbol{q}) \text{ subject to,} \\
&\boldsymbol{R}_m\boldsymbol{q} = \boldsymbol{r}_m, \qquad (1) \\
&\boldsymbol{q}_E \geq 0,
\end{aligned}
$$

where "$\circ$" is the symbol for an element-wise product. In this case, the sum of all q values are minimized. We actually need to minimize the sum of the q_Es. However, in many cases both render the same solution, as will be exemplified later on.

- Based on the resulting q values, calculate the adjusted r_m values by $\boldsymbol{r}_m^{adj} = \boldsymbol{R}_m^{adj}\boldsymbol{q}_F$.

Note that this solution does not imply that $\tilde{\boldsymbol{\eta}}_E = 0$, instead it implies that $\tilde{\boldsymbol{\eta}}_E = \boldsymbol{q}_E$.

This procedure leads to a rewritten hypothesis $H_m^{adj} : \boldsymbol{R}_m^{adj}\boldsymbol{\eta}^{adj} > \boldsymbol{r}_m^{adj}$ (see Equation 4 in the main text). Consequently, the solution for $\boldsymbol{\eta}^{adj}$ renders $\tilde{\boldsymbol{\eta}}_F$. Next, we will provide three examples in each of which either **Step 2(a)**, **Step 2(b)i**, or **Step 2(b)ii** needs to be applied.

An example of **Step 2(a)** in the procedure is provided by evaluating hypothesis

$H_m: \eta_1 > \eta_2, \eta_1 > \eta_3, 2\eta_1 + \eta_2 + \eta_3 > 1$ with $\hat{\eta}_2 = 0$. After discarding the second parameter η_2 (and thus the second column in $\boldsymbol{R}_m$), the rewritten hypothesis is $H_m^{adj}: \eta_1 > 0, \eta_1 > \eta_3, 2\eta_1 + \eta_3 > 1$. Hence, it is implied that $\tilde{\eta}_E = \tilde{\eta}_2$ equals to $\hat{\eta}_2 = 0$. Since $\tilde{\eta}_2 = \hat{\eta}_2 = 0$ is in agreement with the restrictions in H_m, no adjustments are needed in $\boldsymbol{r}_m$:

$$H_m: \boldsymbol{R}_m\boldsymbol{\eta} > \boldsymbol{r}_m \qquad\qquad H_m^{adj}: \boldsymbol{R}_m^{adj}\boldsymbol{\eta}^{adj} > \boldsymbol{r}_m$$

$$\begin{pmatrix} 1 & \cancel{-1} & 0 \\ 1 & \cancel{0} & -1 \\ 2 & \cancel{1} & 1 \end{pmatrix} \begin{pmatrix} \eta_1 \\ \cancel{\eta_2} \\ \eta_3 \end{pmatrix} > \begin{pmatrix} 0 \\ 0 \\ 1 \end{pmatrix} \Longrightarrow \begin{pmatrix} 1 & 0 \\ 1 & -1 \\ 2 & 1 \end{pmatrix} \begin{pmatrix} \eta_1 \\ \eta_3 \end{pmatrix} > \begin{pmatrix} 0 \\ 0 \\ 1 \end{pmatrix}.$$

The concept in **Step 2(b)i** is illustrated by evaluating hypothesis $H_m: \eta_1 > \{\eta_2, \eta_3, \eta_4\}, \eta_2 < 0.3$ with $\hat{\eta}_2 = 0$. After discarding the second parameter and thus the second column in $\boldsymbol{R}_m$, there is a row in $\boldsymbol{R}_m^{adj}$ with only zeros (see the last row in $\boldsymbol{R}_m^{adj}$ below). The rewritten hypothesis is $H_m^{adj}: \eta_1 > 0, \eta_1 > \{\eta_3, \eta_4\}$. Note that the last row in $\boldsymbol{R}_m^{adj}$ below does not impose any restriction on the parameters and the resulting restriction (i.e., $0 \times \eta_1 + 0 \times \eta_3 + 0 \times \eta_4 = 0 > -0.3$) is met. Because $\tilde{\eta}_2 = \hat{\eta}_2 = 0$ is in accordance with the restrictions in H_m (i.e., $0 > -0.3$ holds true), $\boldsymbol{r}_m$ does not change:

$$H_m: \boldsymbol{R}_m\boldsymbol{\eta} > \boldsymbol{r}_m \qquad\qquad H_m^{adj}: \boldsymbol{R}_m^{adj}\boldsymbol{\eta}^{adj} > \boldsymbol{r}_m^{adj}$$

$$\begin{pmatrix} 1 & \cancel{-1} & 0 & 0 \\ 1 & \cancel{0} & -1 & 0 \\ 1 & \cancel{0} & 0 & -1 \\ 0 & \cancel{-1} & 0 & 0 \end{pmatrix} \begin{pmatrix} \eta_1 \\ \cancel{\eta_2} \\ \eta_3 \\ \eta_4 \end{pmatrix} > \begin{pmatrix} 0 \\ 0 \\ 0 \\ -0.3 \end{pmatrix} \Longrightarrow \begin{pmatrix} 1 & 0 & 0 \\ 1 & -1 & 0 \\ 1 & 0 & -1 \\ 0 & 0 & 0 \end{pmatrix} \begin{pmatrix} \eta_1 \\ \eta_3 \\ \eta_4 \end{pmatrix} > \begin{pmatrix} 0 \\ 0 \\ 0 \\ -0.3 \end{pmatrix}.$$

Evaluating hypothesis $H_m: \eta_1 > \{\eta_2, \eta_3, \eta_4\}, \eta_2 > 0.3$ with $\hat{\eta}_2 = 0$ exemplifies **Step 2(b)ii** in the procedure in which not only $\boldsymbol{R}_m$ and $\boldsymbol{\eta}$ but also $\boldsymbol{r}_m$ needs to be adjusted. Discarding the second parameter in H_m renders the false hypothesis $H_m^*: \eta_1 > \{\eta_3, \eta_4\}, \eta_1 > 0.3$ and $0 > 0.3$. In this case, there is a row with only zeros (i.e., the last row) in $\boldsymbol{R}_m^{adj}$, and $\tilde{\eta}_2 = \hat{\eta}_2 = 0$ is not in accordance with the restrictions in H_m, because $0 \times \eta_1 + 0 \times \eta_3 + 0 \times \eta_4 = 0$ is not larger than 0.3:

$$H_m: \boldsymbol{R}_m\boldsymbol{\eta} > \boldsymbol{r}_m \qquad\qquad H_m^*: \boldsymbol{R}_m^{adj}\ \boldsymbol{\eta}^{adj} > \boldsymbol{r}_m^*$$

$$\begin{pmatrix} 1 & -1 & 0 & 0 \\ 1 & 0 & -1 & 0 \\ 1 & 0 & 0 & -1 \\ 0 & 1 & 0 & 0 \end{pmatrix} \begin{pmatrix} \eta_1 \\ \cancel{\eta_2} \\ \eta_3 \\ \eta_4 \end{pmatrix} > \begin{pmatrix} 0 \\ 0 \\ 0 \\ 0.3 \end{pmatrix} \Longrightarrow \begin{pmatrix} 1 & 0 & 0 \\ 1 & -1 & 0 \\ 1 & 0 & -1 \\ 0 & 0 & 0 \end{pmatrix} \begin{pmatrix} \eta_1 \\ \eta_3 \\ \eta_4 \end{pmatrix} > \begin{pmatrix} 0.3 \\ 0 \\ 0 \\ 0.3 \end{pmatrix},$$

where $\boldsymbol{r}_m^*$ contains the constants in hypothesis H_m^*.

Now, $\tilde{\eta}_2$ needs to be calculated in line with the restrictions in H_m. Using **Equation 1**, we obtained $\tilde{\eta}_2 = q_E = 0.3$ and $\boldsymbol{q}_F = (0.3, 0.3, 0.3)$, which leads to:

$$\boldsymbol{r}_m^{adj} = \boldsymbol{R}_m^{adj} \boldsymbol{q}_F = \begin{pmatrix} 1 & 0 & 0 \\ 1 & -1 & 0 \\ 1 & 0 & -1 \\ 0 & 0 & 0 \end{pmatrix} \begin{pmatrix} 0.3 \\ 0.3 \\ 0.3 \end{pmatrix} = \begin{pmatrix} 0.3 \\ 0 \\ 0 \\ 0 \end{pmatrix}.$$

Consequently, the rewritten hypothesis $H_m^{adj} : \boldsymbol{R}_m^{adj} \boldsymbol{\eta}^{adj} > \boldsymbol{r}_m^{adj}$ is described as follows:

$$\underset{H_m \,:\, \boldsymbol{R}_m \boldsymbol{\eta} > \boldsymbol{r}_m}{\begin{pmatrix} 1 & -1 & 0 & 0 \\ 1 & 0 & -1 & 0 \\ 1 & 0 & 0 & -1 \\ 0 & 1 & 0 & 0 \end{pmatrix} \begin{pmatrix} \eta_1 \\ \cancel{\eta_2} \\ \eta_3 \\ \eta_4 \end{pmatrix} > \begin{pmatrix} 0 \\ 0 \\ 0 \\ 0.3 \end{pmatrix}} \Longrightarrow \underset{H_m^{adj} \,:\, \boldsymbol{R}_m^{adj} \boldsymbol{\eta}^{adj} > \boldsymbol{r}_m^{adj}}{\begin{pmatrix} 1 & 0 & 0 \\ 1 & -1 & 0 \\ 1 & 0 & -1 \\ 0 & 0 & 0 \end{pmatrix} \begin{pmatrix} \eta_1 \\ \eta_3 \\ \eta_4 \end{pmatrix} > \begin{pmatrix} 0.3 \\ 0 \\ 0 \\ 0 \end{pmatrix}}.$$

Note that we use $\hat{\eta}_2 = 0.3$ instead of $\tilde{\eta}_2 = \hat{\eta}_2 = 0$ since the latter is not in accordance with the restrictions in H_m. The value of 0.3 results from **Equation 1**, which gives one possible solution regarding the $\tilde{\eta}_E$s for this example.

Sometimes there might be multiple solutions for the order-restricted MLEs, when two or more ηs are estimated as zero. We highlight three such cases. For the first case, the actual solution is irrelevant for the computation of GORICA. For example, for hypothesis $H_m : \eta_1 + \eta_2 + \eta_3 = 0.4, \eta_1 + \eta_2 > 0.3$ with $\hat{\eta}_1 = \hat{\eta}_2 = 0$, **Equation 1** gives $\tilde{\boldsymbol{\eta}}_E = (\tilde{\eta}_{m1}, \tilde{\eta}_{m2})^\top = (0.15, 0.15)^\top$ and based on this solution we obtain $\tilde{\eta}_F = \tilde{\eta}_{m3} = 0.1$. There is actually an infinite number of solutions for $\tilde{\eta}_{m1}$ and $\tilde{\eta}_{m2}$. Stated otherwise, both

$\tilde{\eta}_{m1}$ and $\tilde{\eta}_{m2}$ can take any values ranging from 0 to 0.3 such that their sum is 0.3. All these solutions, irrelevant of the values, give $\tilde{\eta}_F = \tilde{\eta}_{m3} = 0.1$. Hence, in this example, minimizing the sum of squared ηs renders the same solution as minimizing the sum of squared η_Es. For the second case, the choice of the values for $\tilde{\boldsymbol{\eta}}_E$ may influence the results, but the optimal solution is still obtained. For example, for hypothesis $H_m : \eta_1 + \eta_2 + \eta_3 = 0.4, \eta_1 - \eta_2 > 0.3$, there are multiple solutions for $\tilde{\eta}_{m1}$ and $\tilde{\eta}_{m2}$, and these solutions influence the value of $\tilde{\eta}_{m3}$ and, consequently, the results. For example, one possible solution could be $\tilde{\boldsymbol{\eta}}_E = (\tilde{\eta}_{m1}, \tilde{\eta}_{m2})^\top = (0.35, 0.05)^\top$ and, consequently, $\tilde{\eta}_F = \tilde{\eta}_{m3} = 0$ and another possible solution could be $\tilde{\boldsymbol{\eta}}_E = (\tilde{\eta}_{m1}, \tilde{\eta}_{m2})^\top = (0.3, 0)^\top$ and, consequently, $\tilde{\eta}_F = \tilde{\eta}_{m3} = 0.1$. The latter solution is given by **Equation 1** and it is optimal, that is, it gives both the minimal sum of squared $\tilde{\eta}_E$s and the minimal sum of squared $\tilde{\eta}$s. For the third case, **Equation 1** does not give the optimal solution, but it gives a sub-optimal solution, since the sum of squared $\tilde{\eta}_E$s is not minimized, but the sum of all squared $\tilde{\eta}$s is minimized. For example, for hypothesis $H_m : \eta_1 + \eta_2 + \eta_3 = 0.6, \eta_1 - \eta_2 > 0.2$, the optimal solution is $\tilde{\boldsymbol{\eta}}_E = (\tilde{\eta}_{m1}, \tilde{\eta}_{m2})^\top = (0.2, 0)^\top$, and thus, $\tilde{\eta}_F = \tilde{\eta}_{m3} = 0.4$ for which the sum of squared $\tilde{\eta}_E$s is minimal (i.e., $0.2^2 + 0^2 = 0.04$ is the minumum value among all the other solutions). However, **Equation 1** does not give this solution, instead it gives the solution for which the sum of all squared ηs are minimized rendering $\tilde{\boldsymbol{\eta}}_E = (\tilde{\eta}_{m1}, \tilde{\eta}_{m2})^\top = (0.3, 0.1)^\top$, and then, $\tilde{\eta}_F = \tilde{\eta}_{m3} = 0.2$. Note that this last case can only occur when these three conditions hold: (1) two or more ηs are estimated as zero, (2) the hypothesis under evaluation contains a restriction on only these ηs, and (3) $\tilde{\boldsymbol{\eta}}_E = 0$ is not in agreement with this restriction.

Rewriting hypotheses based on $\hat{\eta} = 1$ or η is not estimable

The procedure in the `gorica` package stops in the case of $\hat{\eta} = 1$ due to structural zeros and gives a warning message stating that users should rewrite the hypotheses under evaluation themselves. For example, since $\hat{\eta}_2 = \frac{\hat{\pi}_{12}}{\hat{\pi}_{12} + \hat{\pi}_{22}} = 1$ with $\hat{\pi}_{22} = 0$, $\eta_1 > \eta_2$ can easily be rewritten as $\eta_1 > 1$, which can be evaluated by the `gorica` package. Note that, since

$\hat{\eta}_1 = \frac{\hat{\pi}_{11}}{\hat{\pi}_{11}+\hat{\pi}_{21}} < 1$, hypothesis $H_m : \eta_1 > \eta_2 = 1$ receives no support from the data which can easily be anticipated by the user without evaluating the hypothesis. However, the set of hypotheses in real life applications often consist of more than one hypothesis containing more complicated restrictions on cell probabilities for which rewriting the hypotheses makes more valuable contribution in evaluating the hypotheses.

When some of the η parameters cannot be estimated because of empty cells due to structural zero(s), the package will give an error and suggest rewriting the hypothesis. As an example, hypothesis $H_m : \eta_1 > 1$, with $\eta_1 = \frac{\pi_{11}\pi_{22}}{\pi_{12}\pi_{21}}$ where $\hat{\pi}_{12} = 0$ due to a structural zero, can be inspected equivalently by means of $\eta_1^\diamond = \pi_{11}\pi_{22} - \pi_{12}\pi_{21}$ and $\eta_1^\diamond > 0$. Note that $\hat{\eta}_1^\diamond = \hat{\pi}_{11}\hat{\pi}_{22}$ when $\hat{\pi}_{12} = 0$, which is still estimable and has variation. The parameter $\eta_1^\diamond$ is not contained in Table 2 (in the main text), but is another reparameterization of the same hypothesis in this class. As another example, consider hypothesis $H_m : \eta_1 > \eta_2 > \eta_3$, which is formulated in terms of three local odds ratios with $\boldsymbol{\eta} = (\eta_1, \eta_2, \eta_3)^\top = (\frac{\pi_{11}\pi_{22}}{\pi_{12}\pi_{21}}, \frac{\pi_{12}\pi_{23}}{\pi_{13}\pi_{22}}, \frac{\pi_{13}\pi_{24}}{\pi_{14}\pi_{23}})^\top$, and $\hat{\pi}_{12} = \hat{\pi}_{23} = 0$ because of two empty cells due to structural zeros. In this case, it is not possible to estimate the parameters $\eta_1 = \frac{\pi_{11}\pi_{22}}{\pi_{12}\pi_{21}}$ and $\eta_3 = \frac{\pi_{13}\pi_{24}}{\pi_{14}\pi_{23}}$, and the parameter $\eta_2 = \frac{\pi_{12}\pi_{23}}{\pi_{13}\pi_{22}}$ is estimated as zero. To be able to evaluate this hypothesis, it should be rewritten as $H_m : \eta_1^\diamond > \eta_2^\diamond, \eta_3^\diamond > \eta_4^\diamond$ with $\boldsymbol{\eta}^\diamond = (\eta_1^\diamond, \eta_2^\diamond, \eta_3^\diamond, \eta_4^\diamond)^\top = (\pi_{11}\pi_{13}\pi_{22}^2,\ \pi_{12}^2\pi_{21}\pi_{23},\ \pi_{12}\pi_{14}\pi_{23}^2,\ \pi_{13}^2\pi_{22}\pi_{24})^\top$, and $\hat{\eta}_2^\diamond = \hat{\eta}_4^\diamond = 0$ in the new parameter vector $\boldsymbol{\eta}^\diamond$ because of the aforementioned two empty cells. Thus, hypothesis $H_m : \eta_1^\diamond > \eta_2^\diamond, \eta_3^\diamond > \eta_4^\diamond$ can be rewritten as $H_m : \eta_1^\diamond > 0, \eta_4^\diamond < 0$, which is automated in the `gorica` package. Note that, since all cell probabilities and, consequently, all $\eta^\diamond$s are positive, the restriction $\eta_4^\diamond < 0$ in H_m implies that the corresponding order-restricted MLE is zero, that is, $\tilde{\eta}_4^\diamond = 0$. Note furthermore that we have one parameter more, but we use the same number of restrictions, since $\eta_1^\diamond > \eta_2^\diamond$ and $\eta_3^\diamond > \eta_4^\diamond$ correspond to the restrictions $\eta_1 > \eta_2$ and $\eta_2 > \eta_3$ in hypothesis H_m, respectively. Thus, in some cases hypotheses cannot be directly evaluated by the `gorica` package because of empty cells. Nevertheless, rewriting hypotheses reflecting the same expectations enables researchers to

evaluate the same expectations by the `gorica` package.

The evaluation of hypotheses using our package in the presence of empty cells due to structural zeros is available on the Web at **GitHub**. In the next subsection, we provide a brief guidance on when and how users should rewrite the hypotheses they are interested in.

Guidance on rewriting hypotheses

We provide a step-by-step guidance to `gorica` users in rewriting the hypotheses under consideration, if necessary, with the aid of a summary table. In the summary table, we evaluate hypothesis $H_m : \eta_1 > \eta_2$ when one of the parameters is estimated to be zero or one, or is inestimable due to a structural zero.

1. Check the vector of $\hat{\boldsymbol{\eta}}$ to determine whether it contains zero.
   (a) If there is no zero in $\hat{\boldsymbol{\eta}}$, the hypotheses do not need to be rewritten.
   (b) If $\hat{\boldsymbol{\eta}}$ contains a zero, the hypotheses need to be rewritten by the `gorica` package. Rewriting the hypothesis is fully automized in the `gorica` package for this case and does not require any user intervention (see the first line in **Table 5**).
2. Check the vector of $\hat{\boldsymbol{\eta}}$ to determine whether it contains one and/or is not estimable.
   (a) If $\hat{\boldsymbol{\eta}}$ does not contain one or more 1s and all the parameters are estimable, the hypotheses do not need to be rewritten.
   (b) If $\hat{\boldsymbol{\eta}}$ contains one or more 1s and/or any parameter is not estimable, the hypotheses need to be rewritten. Rewriting the hypothesis is not automized in the `gorica` package for these cases and requires user intervention (see the second and third lines in **Table 5**).

The Eye-Tracking Example for the Second Scenario

This section elaborates on the Eye-Tracking example for the second scenario. In this scenario, the gaze location of Person 2 is considered to influence the gaze location of Person

1. The same hypotheses in (8) in the main text are evaluated which are redisplayed below:

$$\begin{aligned} &H_1: \pi_{11+} > \pi_{1+1},\ \pi_{22+} > \pi_{2+2},\ \pi_{33+} > \pi_{3+3},\ \pi_{44+} > \pi_{4+4}, \\ &H_2: \pi_{11+} > \pi_{1+1},\ \pi_{22+} > \pi_{2+2},\ \pi_{34+} > \pi_{3+4},\ \pi_{43+} > \pi_{4+3}, \\ &H_3: \pi_{11+} = \pi_{1+1},\ \pi_{22+} = \pi_{2+2},\ \pi_{33+} = \pi_{3+3},\ \pi_{44+} = \pi_{4+4}, \\ &H_4: \pi_{11+} = \pi_{1+1},\ \pi_{22+} = \pi_{2+2},\ \pi_{34+} = \pi_{3+4},\ \pi_{43+} = \pi_{4+3}, \\ &H_u: \pi_{11+},\ \pi_{1+1}, ..., \pi_{4+4}. \end{aligned} \tag{2}$$

In **Table 6**, the true cell probabilities and observed cell frequencies are displayed for the second scenario. The estimates of the marginal cell probabilities in (8) in the main text and their covariance matrix for this scenario are given in **Table 7**. The values of the order-restricted maximum log likelihoods and penalties, the GORICA values, and the GORICA weights are displayed for the second scenario in **Table 8**. As can be seen in **Table 8**, hypothesis H_2 is not the only hypothesis supported by the data. Hypotheses H_1 and H_4 also receive some support from the data. Hypotheses H_1, H_2, and H_4 receive $0.314/0.044 \approx 7.14$, $0.268/0.044 \approx 6.09$, and $0.374/0.044 = 8.50$ times more support than the unconstrained hypothesis H_u, respectively. Therefore, H_1 is not considered a weak hypothesis, and it can be meaningfully compared to the other hypotheses. Since the support relative to each other is close to 1, for example, hypothesis H_4 has only $0.374/0.314 \approx 1.19$ and $0.374/0.268 \approx 1.40$ times more support than hypotheses H_1 and H_2, respectively, we conclude that although hypothesis H_4 is the best hypothesis among the four hypotheses, the evidence for this conclusion is not quite obvious.

Evaluating Hypotheses Using the `gorica` Package

This section elaborates on how to use the `gorica` package to evaluate hypotheses for the gender by earned degrees and Eye tracking examples presented in the main text. We set a seed in `R` to reproduce the results in the main text when using the `gorica` package for both examples in this supplementary material. The `R` code in this section are also available

on the Web at **GitHub**.

Example 1: Evaluating non-linear restrictions

First, we elaborate on evaluating hypotheses containing non-linear restrictions on cell probabilities. This problem is solved by reparameterizing cell probabilities in a way that hypotheses contain linear restrictions in the new parameters (see the subsection "Reparameterizing cell probabilities" in the main text). The solution to this problem utilizing the `gorica` package is illustrated by using the gender by earned degrees study presented in the introduction. The data for this example are given in Table 1 (in the main text) which can be entered into `R` as follows:

```
#Use set.seed(111) in R to reproduce the results in Table 9 (in the main text)
set.seed(111)

#Displaying the data on R console
mydata <- as.table(matrix(c(933, 402, 51, 26, 661, 260, 44, 26),
nrow = 2, ncol = 4, byrow = TRUE))
mydata
```

```
    A   B   C   D
A 933 402  51  26
B 661 260  44  26
```

The four hypotheses of interest containing conditional cell probabilities are presented in the main text (see the section "GORICA Illustrated"). The 2×4 contingency table contains $D = 8$ cell probabilities which are denoted by x[i, j], where $i = 1, 2$ and $j = 1, 2, 3, 4$. The hypotheses of interest contain $K = 4$ conditional cell probabilities that are indicated by the functions of the cell probabilities. Note, for example, that x[2, 1] / (x[1, 1] + x[2, 1]) represents the first parameter in $\boldsymbol{\eta} = (\eta_1, \eta_2, \eta_3, \eta_4)^\top = (\frac{\pi_{21}}{\pi_{+1}}, \frac{\pi_{22}}{\pi_{+2}}, \frac{\pi_{23}}{\pi_{+3}}, \frac{\pi_{24}}{\pi_{+4}})^\top$ used for the gender by earned degrees example, that is, $\eta_1 = \frac{\pi_{21}}{\pi_{+1}}$. This is a simple way of defining the functions of cell probabilities in `R`

and flexible in the sense that it is also applicable to high-dimensional contingency tables which will be illustrated later on in this supplementary material. The order-restricted maximum log likelihoods and penalties, the GORICA values, and the GORICA weights for the hypotheses under consideration are obtained as follows:

```
#The order-restricted log likelihoods and penalties, the GORICA values,
#and the GORICA weights for the gender by earned degrees example
res <- gorica(mydata, hypothesis = "a:=x[2,1]/(x[1,1]+x[2,1]);
b:=x[2,2]/(x[1,2]+x[2,2]); c:=x[2,3]/(x[1,3]+x[2,3]);
d:=x[2,4]/(x[1,4]+x[2,4]);
a > (b,c,d); a = b & c > d; a >b & b > c & c > d")
res
```

```
Informative hypothesis test for an object of class table:

   loglik penalty gorica  gorica_weights
H1 9.214  2.584   -13.261 0.238
H2 9.694  2.505   -14.377 0.415
H3 8.544  2.182   -12.724 0.182
Hu 10.267 4.000   -12.535 0.165

Hypotheses:
  H1: a-b>0&a-c>0&a-d>0
  H2: a-b=0&c-d>0
  H3: a-b>0&b-c>0&c-d>0
  Hu: Unconstrained hypothesis
```

The last column in the output above displays the GORICA weights when evaluating these hypotheses simultaneously. The `gorica` package also enables pairwise comparison of the hypotheses under consideration which can be displayed on `R` console as follows:

```
res$relative_weights
```

```
          H1        H2        H3       Hu
H1 1.0000000 0.5721266 1.3075857 1.437435
H2 1.7478649 1.0000000 2.2854831 2.512443
H3 0.7647682 0.4375442 1.0000000 1.099305
Hu 0.6956835 0.3980190 0.9096658 1.000000
```

Example 2: Evaluating hypotheses in the presence of sampling zeros

In this subsection, we elaborate on how to evaluate hypotheses using the `gorica` package in the presence of empty cells due to sampling zeros (see the section "Problem B: Empty Cells due to Sampling Zeros" in the main text). We solved the problem by replacing the zero variances on the diagonal of $\hat{\mathbf{\Sigma}}_{\hat{\eta}}$ with the lowest variance in the same matrix (see the subsection "Adjusting the covariance matrix in the presence of sampling zeros" in the main text). The solution is exemplified by investigating the gaze locations of two persons in two different scenarios. In this subsection, we focus on the first scenario in which the gaze location of Person 1 influences the gaze location of Person 2. An analogous procedure can be used for the second scenario in which the gaze location of Person 2 affects the gaze location of Person 1. The Eye tracking data for the first scenario are given in a three-dimensional contingency table (see Table 10 in the main text). The data are displayed on `R` console as follows:

```
#Use set.seed(111) in R to reproduce the results in Table 12 (in the main text)
set.seed(111)

#Displaying the data on R console
mydata<-as.table(matrix(c(51,2,42,46,7,
4,0,5,3,0,
44,4,174,39,2,
14,3,34,36,4,
4,1,26,11,0,
1,0,1,2,0,
```

```
0,0,0,0,0,
5,0,4,2,0,
1,0,3,0,0,
0,0,0,0,0,
1006,112,921,495,91,
81,1,79,22,2,
1084,109,1909,397,134,
486,42,419,220,33,
63,3,151,26,1,
34,3,7,21,1,
8,0,2,1,0,
23,0,20,13,3,
6,11,5,0,0,
0,0,3,0,0,
28,0,18,4,7,
0,0,2,0,0,
22,2,98,14,1,
5,0,25,0,0,
2,0,0,0,0), nrow=25, ncol=5, byrow = TRUE))
mydata
```

	A	B	C	D	E
A	51	2	42	46	7
B	4	0	5	3	0
C	44	4	174	39	2
D	14	3	34	36	4
E	4	1	26	11	0
F	1	0	1	2	0
G	0	0	0	0	0
H	5	0	4	2	0
I	1	0	3	0	0
J	0	0	0	0	0
K	1006	112	921	495	91
L	81	1	79	22	2
M	1084	109	1909	397	134
N	486	42	419	220	33
O	63	3	151	26	1
P	34	3	7	21	1
Q	8	0	2	1	0
R	23	0	20	13	3
S	6	11	5	0	0
T	0	0	3	0	0
U	28	0	18	4	7
V	0	0	2	0	0
W	22	2	98	14	1
X	5	0	25	0	0
Y	2	0	0	0	0

The five hypotheses containing marginal cell probabilities are introduced in (8) in the main text. These hypotheses contain $K = 12$ marginal cell probabilities and the three-dimensional contingency table contains $D = 125$ cell probabilities (see Table 10 in the main text). The marginal cell probabilities for the $5 \times 5 \times 5$ contingency table are denoted by the letters $a, b, ..., l$, respectively. Even within the context of high-dimensional contingency tables with or without empty cells, the functions of cell probabilities can easily be specified by means of using a two-dimensional space. For example, $\mathrm{x}[1,\ 1] + \mathrm{x}[1,\ 2] + \mathrm{x}[1,\ 3] + \mathrm{x}[1,\ 4] + \mathrm{x}[1,\ 5]$ stands for the first marginal cell probability in $\boldsymbol{\eta} = (\eta_1, \eta_2, ..., \eta_{12})^\top = (\pi_{11+}, \pi_{1+1}, ..., \pi_{4+4})^\top$, where $\eta_1 = \pi_{11+}$. In this way, the data in the

three dimensional contingency table are stacked into a two dimensional parameter space. The order-restricted maximum log likelihoods and penalties, the GORICA values, and the GORICA weights for the hypotheses presented in the Eye tracking example are obtained in the `gorica` package as follows:

```
res <- gorica(mydata, hypothesis = "a:=x[1,1]+x[1,2]+x[1,3]+x[1,4]+x[1,5];
b:=x[1,1]+x[2,1]+x[3,1]+x[4,1]+x[5,1];
c:=x[7,1]+x[7,2]+x[7,3]+x[7,4]+x[7,5];
d:=x[6,2]+x[7,2]+x[8,2]+x[9,2]+x[10,2];
e:=x[13,1]+x[13,2]+x[13,3]+x[13,4]+x[13,5];
f:=x[11,3]+x[12,3]+x[13,3]+x[14,3]+x[15,3];
g:=x[14,1]+x[14,2]+x[14,3]+x[14,4]+x[14,5];
h:=x[11,4]+x[12,4]+x[13,4]+x[14,4]+x[15,4];
i:=x[18,1]+x[18,2]+x[18,3]+x[18,4]+x[18,5];
j:=x[16,3]+x[17,3]+x[18,3]+x[19,3]+x[20,3];
k:=x[19,1]+x[19,2]+x[19,3]+x[19,4]+x[19,5];
l:=x[16,4]+x[17,4]+x[18,4]+x[19,4]+x[20,4];
a > b & c > d & e > f & k > l;
a > b & c > d & g > h & i > j;
a = b & c = d & e = f & k = l;
a = b & c = d & g = h & i = j")

#Displaying the estimates of marginal cell probabilities and
#their covariance matrix
res$estimates

     a      b      c    d      e      f      g      h      i      j      k      l
0.0167 0.0132 0.00 0.00 0.4104 0.3930 0.1355 0.1310 0.0066 0.0041 0.0024 0.0039

res$Sigma
```

	a	b	c	d	e	f	g	h	i	j	k	l
a	1.82-06	6.15e-07	0.00	0.00	-1.12e-06	-5.77e-07	-2.56e-07	-2.30e-07	-1.37e-08	-5.55e-08	1.64e-08	-1.82e-08
b	6.15e-07	1.49e-06	0.00	0.00	-2.88e-07	-4.92e-07	-4.24e-07	-2.50e-07	-4.55e-08	-5.53e-09	-1.01e-08	-2.19e-08
c	0.00	0.00	0.00	0.00	0.00	0.00	0.00	0.00	0.00	0.00	0.00	0.00
d	0.00	0.00	0.00	0.00	0.00	0.00	0.00	0.00	0.00	0.00	0.00	0.00
e	-1.12e-06	-2.88e-07	0.00	0.00	2.78e-05	6.66e-06	-6.39e-06	-1.47e-06	-2.19e-07	-1.96e-08	-1.23e-08	-2.40e-07
f	-5.77e-07	-4.92e-07	0.00	0.00	6.66e-06	2.61e-05	-3.14e-07	-5.29e-06	-2.07e-07	-1.78e-07	-1.07e-07	-2.34e-07
g	-2.56e-07	-4.24e-07	0.00	0.00	-6.39e-06	-3.14e-07	1.33e-05	3.97e-07	2.75e-08	-3.11e-08	3.87e-08	-1.45e-08
h	-2.30e-07	-2.50e-07	0.00	0.00	-1.47e-06	-5.29e-06	3.97e-07	1.22e-05	-1.95e-07	-6.21e-08	-8.95e-08	-1.19e-07
i	-1.37e-08	-4.55e-08	0.00	0.00	-2.19e-07	-2.07e-07	2.75e-08	-1.95e-07	7.22e-07	2.37e-07	8.93e-09	1.56e-07
j	-5.55e-08	-5.53e-09	0.00	0.00	-1.96e-08	-1.78e-07	-3.11e-08	-6.21e-08	2.37e-07	4.52e-07	5.38e-08	-1.15e-09
k	1.64e-08	-1.01e-08	0.00	0.00	-1.23e-08	-1.07e-07	3.87e-08	-8.95e-08	8.93e-09	5.38e-08	3.17e-07	-7.35e-09
l	-1.82e-08	-2.19e-08	0.00	0.00	-2.40e-07	-2.34e-07	-1.45e-08	-1.19e-07	1.56e-07	-1.15e-09	-7.35e-09	4.24e-07

The covariance matrix above contains zero variances on the diagonal due to sampling zeros in the contingency table. Because of the lack of variation, we cannot use the `gorica` package with this covariance matrix, see the explanation in the main text on page 27. In line with our solution presented in the section "Problem B: Empty Cells due to Sampling Zeros" in the main text, we replace the zero variances on the diagonal with the lowest variance in the covariance matrix as follows:

```
#Replacing the zero variances on the diagonal of the covariance matrix with the
#lowest variance in the same matrix
est <- res$estimates
covmtrx <- res$Sigma
diag(covmtrx[c(which(diag(covmtrx == 0))), c(which(diag(covmtrx == 0)))]) =
min(diag(covmtrx)[diag(covmtrx) > 0])

#The values of the likelihood and penalty parts, GORICA values, and GORICA weights
res2 <- gorica(est, Sigma = covmtrx, hypothesis = "a > b & c > d & e > f & k > l;
a > b & c > d & g > h & i > j;
a = b & c = d & e = f & k = l;
a = b & c = d & g = h & i = j")
res2
```

```
Informative hypothesis test for an object of class numeric:

   loglik penalty gorica   gorica_weights
H1 67.228 9.983   -114.490 0.182
H2 68.654 10.044  -117.220 0.712
H3 59.779 8.000   -103.559 0.001
H4 61.481 8.000   -106.962 0.004
Hu 68.654 12.000  -113.308 0.101

Hypotheses:
  H1: a>b&c>d&e>f&k>l
  H2: a>b&c>d&g>h&i>j
  H3: a=b&c=d&e=f&k=l
  H4: a=b&c=d&g=h&i=j
  Hu: Unconstrained hypothesis
```

References

Soetaert, K., Van den Meersche, K., & Van Oevelen, D. (2014). limSolve: Solving linear inverse models. *R Package Version 1.5.5.1.* Retrieved from **https://CRAN.R-project.org/package=limSolve**

Table 1

Calculation of the penalty (weights) for hypotheses containing non-marginal cell probabilities in terms of πs based on $c\hat{\boldsymbol{\Sigma}}_{\hat{\boldsymbol{\pi}}}$ and ηs based on $c\hat{\boldsymbol{\Sigma}}_{\hat{\boldsymbol{\eta}}}$

	$c = 1$		$c = 100000$	
Hypothesis	PT_m	w_m^P	PT_m	w_m^P
H_1: $\pi_{11} - \pi_{21} > \pi_{14} - \pi_{24}$	6.531	**0.615**	2.562	0.442
H_u: $\pi_{11}, \pi_{12}, ..., \pi_{24}$	7.000	**0.385**	2.328	0.558
	PT_m	w_m^P	PT_m	w_m^P
H_1: $\eta_1 - \eta_2 > \eta_3 - \eta_4$	3.499	**0.623**	3.499	**0.623**
H_u: $\eta_1, \eta_2, \eta_3, \eta_4$	4.000	**0.377**	4.000	**0.377**
Hypothesis	PT_m	w_m^P	PT_m	w_m^P
H_2: $\pi_{21} > \pi_{22} > \pi_{23} > \pi_{24}$	5.173	**0.861**	2.455	0.468
H_u: $\pi_{11}, \pi_{12}, ..., \pi_{24}$	7.000	**0.139**	2.328	0.532
	PT_m	w_m^P	PT_m	w_m^P
H_2: $\eta_1 > \eta_2 > \eta_3 > \eta_4$	2.113	**0.868**	2.117	**0.868**
H_u: $\eta_1, \eta_2, \eta_3, \eta_4$	4.000	**0.132**	4.000	**0.132**

Note. $\boldsymbol{\eta} = (\eta_1, \eta_2, \eta_3, \eta_4)^\top = (\pi_{11}, \pi_{14}, \pi_{21}, \pi_{24})^\top$ for hypothesis H_1 and $\boldsymbol{\eta} = (\eta_1, \eta_2, \eta_3, \eta_4)^\top = (\pi_{21}, \pi_{22}, \pi_{23}, \pi_{24})^\top$for hypothesis H_2. The bold text indicates the penalty weights that are close to each other.

Table 2

Calculation of the penalty (weights) for hypotheses containing marginal cell probabilities in terms of πs based on $c\hat{\mathbf{\Sigma}}_{\hat{\pi}}$ and ηs based on $c\hat{\mathbf{\Sigma}}_{\hat{\eta}}$

	$c = 1$		$c = 100000$	
Hypothesis	PT_m	w_m^P	PT_m	w_m^P
H_1: $\pi_{+1} > \{\pi_{+2}, \pi_{+3}, \pi_{+4}\}$	6.338	**0.660**	3.096	0.317
H_u: $\pi_{11}, \pi_{12}, ..., \pi_{24}$	7.000	**0.340**	2.328	0.683
	PT_m	w_m^P	PT_m	w_m^P
H_1: $\eta_1 > \{\eta_2, \eta_3, \eta_4\}$	2.250	**0.679**	2.249	**0.679**
H_u: $\eta_1, \eta_2, \eta_3, \eta_4$	3.000	**0.321**	3.000	**0.321**
Hypothesis	PT_m	w_m^P	PT_m	w_m^P
H_2: $\pi_{1+} > \pi_{2+}$	6.498	**0.623**	3.089	0.318
H_u: $\pi_{11}, \pi_{12}, ..., \pi_{24}$	7.000	**0.377**	2.328	0.682
	PT_m	w_m^P	PT_m	w_m^P
H_2: $\eta_1 > \eta_2$	0.498	**0.623**	0.498	**0.623**
H_u: η_1, η_2	1.000	**0.377**	1.000	**0.377**

Note. $\boldsymbol{\eta} = (\eta_1, \eta_2, \eta_3, \eta_4)^\top = (\pi_{+1}, \pi_{+2}, \pi_{+3}, \pi_{+4})^\top$ for hypothesis H_1 and $\boldsymbol{\eta} = (\eta_1, \eta_2)^\top = (\pi_{1+}, \pi_{2+})^\top$ for hypothesis H_2. The bold text indicates the penalty weights that are close to each other.

Table 3

Calculation of the penalty (weights) for hypotheses containing conditional cell probabilities in terms of πs *based on* $c\hat{\mathbf{\Sigma}}_{\hat{\boldsymbol{\pi}}}$ *and* ηs *based on* $c\hat{\mathbf{\Sigma}}_{\hat{\boldsymbol{\eta}}}$

	$c = 1$		$c = 100000$	
Hypothesis	PT_m	w_m^P	PT_m	w_m^P
H_1: $\frac{\pi_{21}}{\pi_{+1}} > \{\frac{\pi_{22}}{\pi_{+2}}, \frac{\pi_{23}}{\pi_{+3}}, \frac{\pi_{24}}{\pi_{+4}}\}$	6.322	0.663	0.884	**0.809**
H_u: $\pi_{11}, \pi_{12}, ..., \pi_{24}$	7.000	0.337	2.328	**0.191**
	PT_m	w_m^P	PT_m	w_m^P
H_1: $\eta_1 > \{\eta_2, \eta_3, \eta_4\}$	2.547	**0.810**	2.547	**0.810**
H_u: $\eta_1, \eta_2, \eta_3, \eta_4$	4.000	**0.190**	4.000	**0.190**
Hypothesis	PT_m	w_m^P	PT_m	w_m^P
H_2: $\frac{\pi_{12}}{\pi_{1+}} > \frac{\pi_{22}}{\pi_{2+}}$	6.559	**0.608**	1.753	0.640
H_u: $\pi_{11}, \pi_{12}, ..., \pi_{24}$	7.000	**0.392**	2.328	0.360
	PT_m	w_m^P	PT_m	w_m^P
H_2: $\eta_1 > \eta_2$	1.501	**0.622**	1.501	**0.622**
H_u: η_1, η_2	2.000	**0.378**	2.000	**0.378**

Note. $\boldsymbol{\eta} = (\eta_1, \eta_2, \eta_3, \eta_4)^\top = (\frac{\pi_{21}}{\pi_{+1}}, \frac{\pi_{22}}{\pi_{+2}}, \frac{\pi_{23}}{\pi_{+3}}, \frac{\pi_{24}}{\pi_{+4}})^\top$ for hypothesis H_1 and $\boldsymbol{\eta} = (\eta_1, \eta_2)^\top = (\frac{\pi_{12}}{\pi_{1+}}, \frac{\pi_{22}}{\pi_{2+}})^\top$ for hypothesis H_2. The bold text indicates the penalty weights that are close to each other.

Table 4

Calculation of the penalty (weights) for hypotheses containing local odds ratios in terms of πs based on $c\hat{\boldsymbol{\Sigma}}_{\hat{\boldsymbol{\pi}}}$ and ηs based on $c\hat{\boldsymbol{\Sigma}}_{\hat{\boldsymbol{\eta}}}$

	$c = 1$		$c = 100000$	
Hypothesis	PT_m	w_m^P	PT_m	w_m^P
H_1: $\frac{\pi_{11}\pi_{22}}{\pi_{12}\pi_{21}} = 1, \frac{\pi_{13}\pi_{24}}{\pi_{14}\pi_{23}} > 1$	5.540	**0.812**	1.052	0.782
H_u: $\pi_{11}, \pi_{12}, ..., \pi_{24}$	7.000	**0.188**	2.328	0.218
	PT_m	w_m^P	PT_m	w_m^P
H_1: $\eta_1 = 1, \eta_2 > 1$	0.501	**0.817**	0.501	**0.817**
H_u: η_1, η_2	2.000	**0.183**	2.000	**0.183**
Hypothesis	PT_m	w_m^P	PT_m	w_m^P
H_2: $\frac{\pi_{12}\pi_{23}}{\pi_{13}\pi_{22}} > \frac{\pi_{13}\pi_{24}}{\pi_{14}\pi_{23}} > 1$	5.877	**0.755**	1.284	0.740
H_u: $\pi_{11}, \pi_{12}, ..., \pi_{24}$	7.000	**0.254**	2.328	0.260
	PT_m	w_m^P	PT_m	w_m^P
H_2: $\eta_1 > \eta_2 > 1$	0.627	**0.798**	0.627	**0.798**
H_u: η_1, η_2	2.000	**0.202**	2.000	**0.202**

Note. $\boldsymbol{\eta} = (\eta_1, \eta_2)^\top = (\frac{\pi_{11}\pi_{22}}{\pi_{12}\pi_{21}}, \frac{\pi_{13}\pi_{24}}{\pi_{14}\pi_{23}})^\top$ for hypothesis H_1 and $\boldsymbol{\eta} = (\eta_1, \eta_2)^\top = (\frac{\pi_{12}\pi_{23}}{\pi_{13}\pi_{22}}, \frac{\pi_{13}\pi_{24}}{\pi_{14}\pi_{23}})^\top$ for hypothesis H_2. The bold text indicates the penalty weights that are close to each other.

Table 5

Summary table on rewriting $H_m : \eta_1 > \eta_2$ in the presence of an empty cell due to a structural zero

Parameters	Problematic estimate	Rewritten hypothesis	User inter.
$\boldsymbol{\eta} = (\eta_1, \eta_2)^\top = (\frac{\pi_{11}}{\pi_{11}+\pi_{21}}, \frac{\pi_{12}}{\pi_{12}+\pi_{22}})^\top$, with $\hat{\pi}_{12} = 0$	$\hat{\eta}_2 = 0$	$H_m : \eta_1 > 0$	No
$\boldsymbol{\eta} = (\eta_1, \eta_2)^\top = (\frac{\pi_{11}}{\pi_{11}+\pi_{21}}, \frac{\pi_{12}}{\pi_{12}+\pi_{22}})^\top$, with $\hat{\pi}_{21} = 0$	$\hat{\eta}_1 = 1$	$H_m : \eta_2 < 1$	Yes
$\boldsymbol{\eta} = (\eta_1, \eta_2)^\top = (\frac{\pi_{11}\pi_{22}}{\pi_{12}\pi_{21}}, \frac{\pi_{12}\pi_{23}}{\pi_{22}\pi_{13}})^\top$, with $\hat{\pi}_{21} = 0$	η_1 is not estimable	$H_m : \pi_{11}\pi_{22} - \pi_{22}\pi_{13} > 0$	Yes

Table 6

Population probabilities π_{ijv} and observed cell frequencies between brackets for the eye-tracking example in the case of the gaze location of Person 2 influences the gaze location of Person 1

π_{ijv}		Person 1 at time point $t+200$				
Person 2 at time point t	Person 1 at time point $t+20$	Nose	Mouth	Right Eye	Left Eye	None
1 = Nose	Nose	π_{111} (5)	π_{112} (0)	π_{113} (155)	π_{114} (3)	π_{115} (9)
	Mouth	π_{121} (0)	π_{122} (0)	π_{123} (10)	π_{124} (0)	π_{125} (0)
	Right Eye	π_{131} (124)	π_{132} (5)	π_{133} (2350)	π_{134} (38)	π_{135} (82)
	Left Eye	π_{141} (5)	π_{142} (3)	π_{143} (47)	π_{144} (0)	π_{145} (1)
	None	π_{151} (5)	π_{152} (0)	π_{153} (61)	π_{154} (0)	π_{155} (2)
2 = Mouth	Nose	π_{211} (1)	π_{212} (0)	π_{213} (8)	π_{214} (0)	π_{215} (0)
	Mouth	π_{221} (0)	π_{222} (0)	π_{223} (0)	π_{224} (0)	π_{225} (0)
	Right Eye	π_{231} (14)	π_{232} (0)	π_{233} (162)	π_{234} (15)	π_{235} (6)
	Left Eye	π_{241} (0)	π_{242} (0)	π_{243} (2)	π_{244} (1)	π_{245} (0)
	None	π_{251} (1)	π_{252} (0)	π_{253} (1)	π_{254} (0)	π_{255} (0)
3 = Right Eye	Nose	π_{311} (14)	π_{312} (2)	π_{313} (264)	π_{314} (4)	π_{315} (9)
	Mouth	π_{321} (1)	π_{322} (0)	π_{323} (8)	π_{324} (0)	π_{325} (0)
	Right Eye	π_{331} (365)	π_{332} (47)	π_{333} (3009)	π_{334} (78)	π_{335} (86)
	Left Eye	π_{341} (4)	π_{342} (4)	π_{343} (69)	π_{344} (8)	π_{345} (1)
	None	π_{351} (10)	π_{352} (0)	π_{353} (122)	π_{354} (4)	π_{355} (0)
4 = Left Eye	Nose	π_{411} (3)	π_{412} (0)	π_{413} (46)	π_{414} (0)	π_{415} (1)
	Mouth	π_{421} (0)	π_{422} (0)	π_{423} (0)	π_{424} (0)	π_{425} (0)
	Right Eye	π_{431} (45)	π_{432} (2)	π_{433} (1154)	π_{434} (13)	π_{435} (15)
	Left Eye	π_{441} (6)	π_{442} (14)	π_{443} (9)	π_{444} (0)	π_{445} (1)
	None	π_{451} (1)	π_{452} (0)	π_{453} (17)	π_{454} (1)	π_{455} (0)
5 = None	Nose	π_{511} (5)	π_{512} (0)	π_{513} (27)	π_{514} (0)	π_{515} (0)
	Mouth	π_{521} (0)	π_{522} (0)	π_{523} (0)	π_{524} (0)	π_{525} (0)
	Right Eye	π_{531} (15)	π_{532} (0)	π_{533} (214)	π_{534} (20)	π_{535} (8)
	Left Eye	π_{541} (0)	π_{542} (0)	π_{543} (0)	π_{544} (5)	π_{545} (1)
	None	π_{551} (3)	π_{552} (0)	π_{553} (0)	π_{554} (0)	π_{555} (0)

Table 7

The estimates $\hat{\boldsymbol{\eta}}$ and the adjusted covariance matrix $\hat{\boldsymbol{\Sigma}}_{\hat{\boldsymbol{\eta}}}^{adj}$ for the eye-tracking example

		In the case of the gaze location of Person 2 influences the gaze location Person 1											
		$\hat{\boldsymbol{\Sigma}}_{\hat{\boldsymbol{\eta}}}$											
$\boldsymbol{\eta}$	$\hat{\boldsymbol{\eta}}$	η_1	η_2	η_3	η_4	η_5	η_6	η_7	η_8	η_9	η_{10}	η_{11}	η_{12}
η_1	0.019	2.2e-6											
η_2	0.016	-7.8e-8	1.7e-6										
η_3	0.000	0.000	0.000	1.7e-7									
η_4	0.000	0.000	0.000	0.000	1.7e-7								
η_5	0.405	-9.7e-7	-5.6e-7	0.000	0.000	2.5e-5							
η_6	0.392	-1.1e-6	-3.9e-7	0.000	0.000	1.9e-5	2.6e-5						
η_7	0.010	-8.1e-8	3.7e-8	0.000	0.000	-2.5e-7	5.2e-7	1.1e-6					
η_8	0.011	4.0e-8	-8.4e-9	0.000	0.000	6.0e-7	-4.4e-7	1.1e-7	1.1e-6				
η_9	0.139	-1.3e-7	-4.2e-7	0.000	0.000	-5.8e-6	-5.5e-6	-2.0e-7	-2.8e-7	1.3e-5			
η_{10}	0.139	-1.9e-7	-4.0e-7	0.000	0.000	-5.8e-6	-5.2e-6	-1.6e-7	-3.5e-7	1.3e-5	1.4e-5		
η_{11}	0.003	8.4e-9	-5.6e-8	0.000	0.000	-6.0e-9	-3.7e-8	-5.5e-9	-1.8e-8	2.5e-8	1.5e-7	3.6e-7	
η_{12}	0.002	1.9e-9	-2.2e-8	0.000	0.000	5.9e-9	2.3e-8	-1.1e-8	-1.9e-8	5.7e-8	-7.5e-8	-1.1e-8	1.7e-7

Note. $\boldsymbol{\eta} = (\eta_1, \eta_2, \eta_3, \eta_4, \eta_5, \eta_6, \eta_7, \eta_8, \eta_9, \eta_{10}, \eta_{11}, \eta_{12})^\top = (\pi_{11+}, \pi_{1+1}, \pi_{22+}, \pi_{2+2}, \pi_{33+}, \pi_{3+3}, \pi_{34+}, \pi_{3+4}, \pi_{43+}, \pi_{4+3}, \pi_{44+}, \pi_{4+4})^\top$.

Table 8

The order-restricted maximum log likelihoods $L(\tilde{\boldsymbol{\eta}}_m|\hat{\boldsymbol{\eta}}, \hat{\boldsymbol{\Sigma}}_{\hat{\boldsymbol{\eta}}}^{adj})$*, the penalties* $PT_m(\boldsymbol{\eta})$*, the GORICA values* $GORICA_m$*, and the GORICA weights* w_m *for hypothesis* H_m *with* $m = 1, 2, 3, 4,$ *and u in the case of the gaze location of Person 2 influences the gaze location Person 1*

H_m	$L(\tilde{\boldsymbol{\eta}}_m\|\hat{\boldsymbol{\eta}}, \hat{\boldsymbol{\Sigma}}_{\hat{\boldsymbol{\eta}}}^{adj})$	$PT_m(\boldsymbol{\eta})$	$GORICA_m$	w_m
H_1	70.089	10.024	-120.130	0.314
H_2	69.877	9.972	-119.810	0.268
H_3	59.876	8.000	-103.753	0.000
H_4	68.238	8.000	-120.475	0.374
H_u	70.089	12.000	-116.178	0.044
